# Examining Neanderthal and carnivore occupations of Teixoneres Cave (Moià, Barcelona, Spain) using archaeostratigraphic and intra-site spatial analysis

**DOI:** 10.1038/s41598-021-83741-9

**Published:** 2021-02-22

**Authors:** Leandro Zilio, Heidi Hammond, Theodoros Karampaglidis, Laura Sánchez-Romero, Ruth Blasco, Florent Rivals, Anna Rufà, Andrea Picin, M. Gema Chacón, Martina Demuro, Lee J. Arnold, Jordi Rosell

**Affiliations:** 1 Consejo Nacional de Investigaciones Científicas y Técnicas (CONICET), Universidad Nacional de la Patagonia “San Juan Bosco”, Facultad de Humanidades y Cs. Sociales, 9200 Esquel, Argentina; 2 MONREPOS, Archaeological Research Centre and Museum for Human Behavioural Evolution, Schloss Monrepos, 56567 Neuwied, Germany; 3 Human Evolution Research Center, University of California, 3101 Valley Life Sciences Building, Berkeley, 94720 USA; 4 Institut Català de Paleoecologia Humana i Evolució Social (IPHES-CERCA), Zona Educacional 4, Campus Sescelades URV (Edifici W3), 43007 Tarragona, Spain; 5 Departament d’Història i Història de l’Art, Universitat Rovira i Virgili (URV), 43002 Tarragona, Spain; 6 ICREA, 08010 Barcelona, Spain; 7 University of Bordeaux, CNRS, MCC, PACEA, UMR 5199, 33600 Pessac, France; 8 Department of Human Evolution, Max Planck Institute for Evolutionary Anthropology, Deutscher Platz 6, 04103 Leipzig, Germany; 9 UMR7194 Histoire Naturelle de l’Homme Préhistorique (HNHP), Museum National d’Histoire Naturelle (MNHN), CNRS, Université Perpignan Via Domitia, Alliance Sorbonne Université-Musée de l’Homme, Place du Trocadéro 17, 75016 Paris, France; 10 School of Physical Sciences, Environment Institute, and Institute for Photonics and Advanced Sensing (IPAS), University of Adelaide, North Terrace Campus, Adelaide, 5005 Australia

**Keywords:** Archaeology, Cultural evolution

## Abstract

Teixoneres Cave (Moià, Barcelona, Spain) is a reference site for Middle Palaeolithic studies of the Iberian Peninsula. The cave preserves an extensive stratigraphic sequence made up of eight units, which is presented in depth in this work. The main goal of this study is to undertake an initial spatial examination of Unit III, formed during Marine Isotope Stage 3, with the aim of understanding spatial organization and past activities developed by Neanderthals and carnivores (bears, hyenas and smaller carnivores). The total sample analysed includes 38,244 archaeological items and 5888 limestone blocks. The application of GIS tools allows us to clearly distinguish three geologically-defined stratigraphic subunits. Unit III has been previously interpreted as a palimpsest resulting from alternating occupation of the cave by human groups and carnivores. The distribution study shows that faunal specimens, lithic artefacts, hearths and charcoal fragments are significantly concentrated at the entrance of the cave where, it is inferred, hominins carried out different activities, while carnivores preferred the sheltered zones in the inner areas of the cave. The results obtained reveal a spatial pattern characterized by fire use related zones, and show that the site was occupied by Neanderthals in a similar and consistent way throughout the ˃ 7000 years range covered by the analysed subunits. This spatial pattern is interpreted as resulting from repeated short-term human occupations.

## Introduction

Many European Middle and Late Pleistocene archaeological sites in caves and rock shelters preserve evidence of alternating human and carnivore occupations. These complex occupation dynamics frequently produce overlapping assemblages (palimpsests) due to low sedimentation rates and complex post-depositional processes. The conditions of these palimpsests commonly cause interpretative problems for archaeologists as the constituent assemblages cannot be easily disaggregated into individual components^1–12^; effectively they represent mixing of materials from different and discrete events made by different actors on the same surface of a confined area^3,13^. Nevertheless, these assemblages can be analysed using a diverse range of modern methodological approaches, and it is often possible to ascertain spatial patterns in the distribution of archaeological items^3,14–16^.

Different taphonomic processes can act to homogenize intra-site distributions of materials in cave and rock shelter settings. Post-depositional processes, such as sediment movements or water flows, can cause the mobilization of materials from their original position of abandonment^2,17^. Animal burrowing can also contribute to the homogenization of cultural deposits^18,19^, including the blurring of discrete features, activity areas and strata. The final outcomes are a loss of data and resolution, which make it difficult to conduct reliable archaeological reconstructions^20,21^. Carnivores not only destroy and modify bones through consumption, but they can also affect the original position of the remains, significantly altering the spatial distribution of materials produced by human activities^18,19,22–25^. Carnivores often aid the formation of palimpsests, especially at sites where recurrent human occupations are interspersed with visits by scavengers^26,27^.

Systematic archaeological research at Teixoneres began in 2003, yielding important archaeological, paleontological, geological and taphonomic data. Unit III has been interpreted as a carnivore den/refuge during MIS 3, in which short-term human occupations took place^11,28–33^. This stratigraphic unit has been described as a palimpsest characterized by an apparently uniformly distributed remains, which accumulated as a result of repeated and variable occupation episodes, mainly by hominins and carnivores (bears, hyenas and other smaller carnivores), low sedimentation rates and the action of natural and anthropogenic formation processes^11^.

The aim of this article is to describe spatial distribution and archaeostratigraphic analyses of remains recovered from Unit III of Teixoneres Cave (MIS 3; Moià, Barcelona, Spain). These materials have been selected due to their significance for understanding Neanderthal and carnivore occupations of the cave, during the late Middle Palaeolithic. We examine the integrity of the site using both the archaeostratigraphic analysis and spatial distribution patterns of artefact and faunal remains, as well as their relation with archaeological features, such as hearths. Using this combined approach, we test the working hypothesis that if the site was occupied recurrently over time and in a consistent or organized manner by human groups, it should be possible to recognise specific spatial patterns in the distribution of the archaeological remains, due to the evidence frequently representing long-term trends or average tendencies rather than discrete occupations^3,14,15,34^. In this sense, the archaeological expectation is to recognise a spatial pattern in the evidences left by humans at the archaeological site, whereby differential distribution of materials could be distinguished by well-delimited accumulations^16,26,35–39^. Additionally, we expect that carnivores were capable of modifying the anthropic space, thus altering the spatial distribution of remains left by human groups^18,22^. Our detailed stratigraphic analysis of the Teixoneres Cave archaeosedimentary sequence, together with examination of the distributional patterns of remains, and the agents and process that could have acted in the formation of the assemblages, allow us to better understand the spatial organization and the activities developed by humans and carnivores in the cave.

## Teixoneres Cave

**Figure 1:**
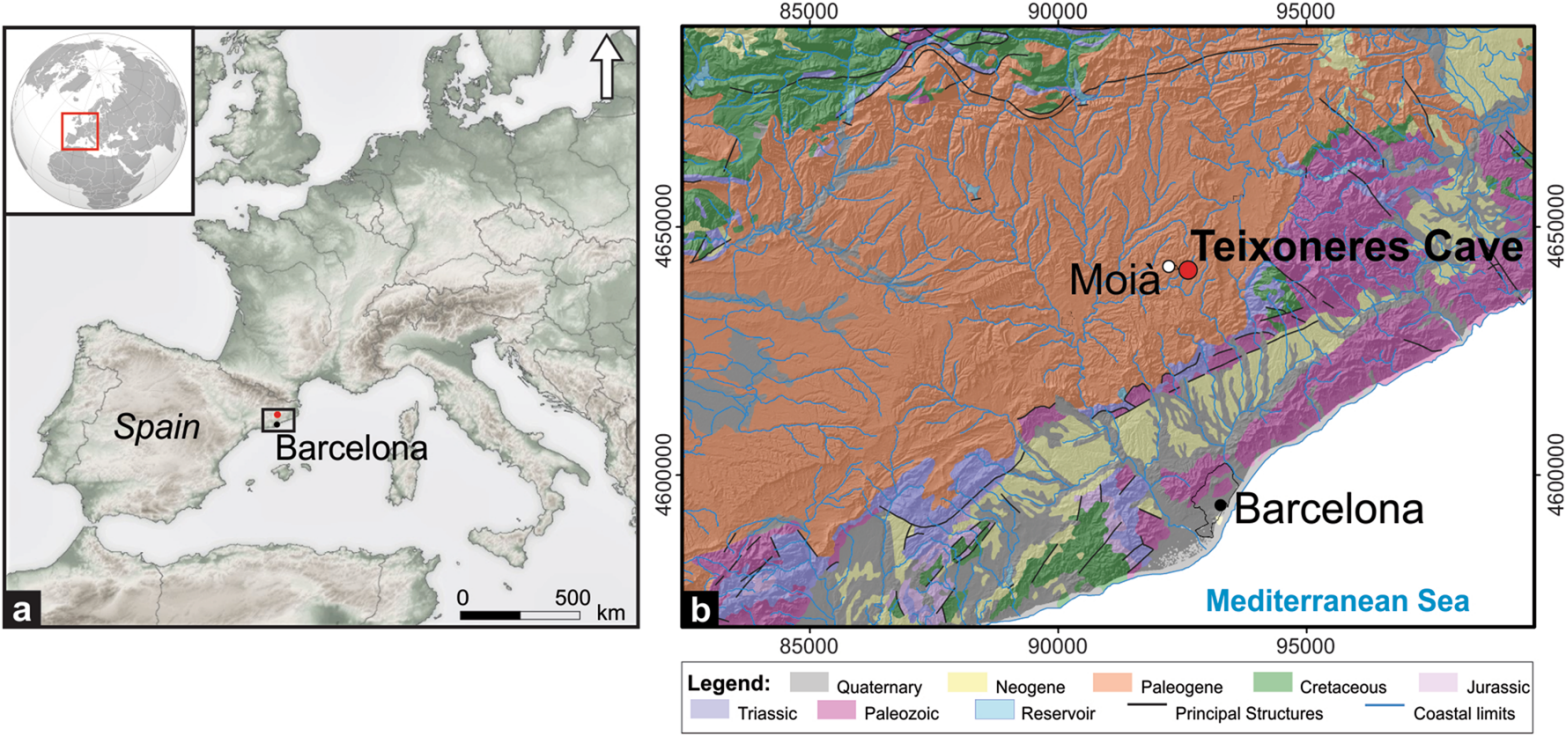
(**a**) Location of Teixoneres Cave. (**b**) Location of Teixoneres Cave and simplified regional geological map of the Moià area. The maps were obtained from an open access source of the Instituto Geológico y Minero de España (IGME), (June 10, 2020) [https://info.igme.es/cartografiadigital/geologica/Geologicos1MMapa.aspx?Id=Geologico1000_(1994)&language=es], and prepared with the Adobe Photoshop CS5 Version 12.0.4 software.

Teixoneres Cave is located in Catalonia, near the town of Moià (Barcelona, Spain) at an altitude of about 760 m a.s.l (Fig. 1). From a geomorphological point of view, the region forms part of the highlands located between two main rivers, the Llobregat to the south and the Ter to the north, which connect the inner area of Northern Catalonia with the Mediterranean Sea. A local speleological group discovered Teixoneres Cave as an archaeological site during the 1950s. In the wake of this discovery, several seasons of archaeological research were carried out, the most significant of which were conducted during the first half of the 1950s and the first half of the 1970s when different pits were excavated^11,40–44^.

The lithic assemblage from Teixoneres shows the knapping of local quartz pebbles for *tranche de saucisson* and discoid *débitage*, and the transport of artefacts in chert and other rocks (metamorphic, sedimentary, and igneous) using Levallois, hierarchized and simple methods. The high fragmentation of the operative chains of the transported artefacts supports the hypothesis of high mobility of Middle Palaeolithic human groups in this territory that recurrently visited the site^11,33,45^. Lithic assemblages are mainly composed by flakes, although retouched elements are also common. Among them, side-scrapers and Mousterian points are the most abundant, although some denticulates are recognized as well. From a technological point of view, the lithic assemblages of Teixoneres are concordant with other Middle Palaeolithic techno-complexes in the region^46^.

The high vertebrate diversity recorded in Unit III suggests an environment marked by a balanced predator–prey dynamic, which may have been interrupted by the occasional presence of small human groups^11,28,29,31^. Red deer (*Cervus elaphus*) is the most abundant species in the faunal assemblage, although other mammals have also been identified, such as *Ursus spelaeus*, *Canis lupus*, *Vulpes vulpes*, *Lynx* sp., *Crocuta crocuta*, *Meles meles*, *Capreolus capreolus*, *Bos primigenius*, *Rupicapra pyrenaica*, *Capra pyrenaica*, *Sus scrofa*, *Equus ferus,* and *Equus hydruntinus*. Unit III is especially interesting due to the occurrence of cold adapted taxa *Mammuthus primigenius* and *Coelodonta antiquitatis*^32,47^. In the case of the leporids, there is clear evidence that these mammals were occasionally consumed at the site by human groups^30^, something that has not been observed in the different bird specimens documented at the site^48^. The ungulate assemblage from the top of this unit indicates cold and arid environmental conditions, and a diverse landscape complex with steppe-like environments, open forested areas, and mountains in the surroundings of the site^32^.

Previous taphonomic studies at Teixoneres showed a clear dichotomy in the use of the cave by human groups and carnivores^11,28^. Carnivores mainly used the innermost galleries and hidden nooks as dens, while the human groups usually settled at the entrance of the cave. According to these observations, no anthropogenic materials have been recovered in the innermost part of the cave and, therefore, the present work is only focused on the outer area and on the southern part of the inner area. Here, we explore that proposition in greater detail by combining information from multiple classes of archaeological data in both areas and testing patterns in their distribution through each subunit.

## Geological setting

**Figure 2:**
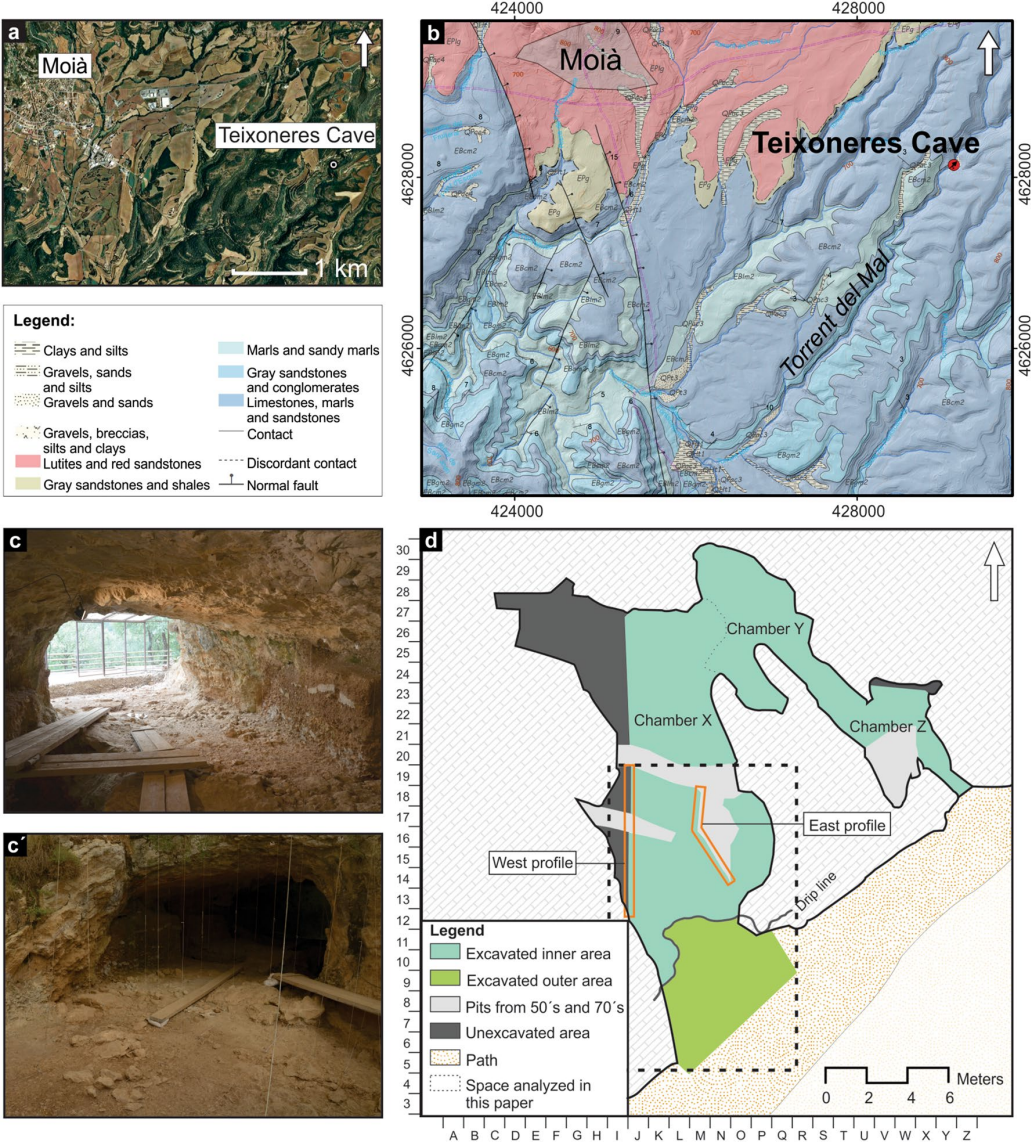
(**a**) Geographic location of Teixoneres Cave. (**b**) Geological map showing the study region. The maps were obtained from an open access source of the Institut Cartogràfic i Geològic de Catalunya (ICGC), (**a**) [https://www.icgc.cat/Descarregues/Imatges-aeries-i-de-satel-lit/Ortoimatges-Sentinel-2] and (**b**) [https://www.icgc.cat/Administracio-i-empresa/Descarregues/Cartografia-geologica-i-geotematica/Cartografia-geologica/GT-I.-Mapa-geologic-1-25.000], used under a CC BY 4.0 license. (**c**–**c′**) View of the archaeological site during the 2017 field season. (**d**) The ground plan and grid system of the excavation was registered by means of a Trimble S6 total station (GPS Trimble R6 and GPS TwoNavSportiva receptors) and using VRS (Virtual References Station) technique. The images were prepared with the Adobe Photoshop CS5 Version 12.0.4 software.

The formation of Teixoneres cave is related with the drainage system of the Torrent del Mal river, and has resulted from the evolution of a karst system developed in Paleogene limestone (Figs. 1 and 2). The cave, located ~ 15 m above from the current floodplain, shows U-shaped morphology with an approximate length of 30 m. It was divided by Serrat and Albert^40^ into three different chambers, named as X, Y and Z.

Chambers X and Z contain entrances to the cave exterior, and are connected by Chamber Y. Chamber X, the biggest gallery, which is 30 m long and 5–6 m wide, is located in the western area and constitutes the main entrance to the cave. This chamber forms a north–south oriented gallery (Fig. 2d).

The cave is filled by a 6-m-thick sediment package, for which different stratigraphic units have been described. The first stratigraphic studies of Teixoneres Cave started in the 1950s by Serra-Ràfols et al*.*^41^, who defined 15 units in Chamber X. This work was continued by Serrat and Albert^40^, who focused on a pit in Chamber X (current squares N–O/14–15) and defined ten lithostratigraphic units. Subsequently, Rosell et al.^11^ using the same pit as Serrat and Albert^40^ defined ten units (I, II, III, IV, V, VI, VII, VIII, IX and X) and four subunits (IIa, IIb, IIIa and IIIb). Finally, Talamo et al*.*^49^ described the upper part of the sequence through a new stratigraphic profile (squares J–K/13–16; Fig. 2d) and redefined four lithostratigraphic units (I–IV) and two subunits for Unit II and III respectively (II-a, II-b, III-a and III-b). These previous geological studies^29,40,41,49^ and extensive excavation of the site, considering stratigraphic, paleontological and archaeological criteria, have allowed us to define in this work eight lithostratigraphic units and 18 subunits: Unit I (I and I-a1); Unit II (II-a1, II-a2, II-b1, II-b2 and II-b3); Unit III (III-a1, III-a2, III-b1, III-b2, III-c, III-d); Unit IV; Unit V (V-a, V-b and V-c); Unit VI (VI-a and VI-b); Unit VII and Unit VIII (Supplementary Sect. 1). Table 1 shows the correlation between the different stratigraphic units defined in previous research and in this work.

**Table 1:** Correlation between the units and subunits defined by Serra-Ràfols et al.^41^, Serrat and Albert^40^, Rosell et al*.*^29^, Talamo et al.^49^, and this paper.

Serra-Ràfols et al.^41^	Serrat and Albert^40^	Rosell et al.^29^	Talamo et al.^49^	This work
I	0	I	I	I
				I-a1
	1	II-a	II-a	II-a1
				II-a2
II	2	II-b	II-b	II-b1
III				II-b2
IV				
V	3 Local	–	–	–
	4	III-a	III-a	III-a1
VI				III-a2
		III-b	III-b	III-b1
VII				III-b2
VIII				III-c
IX				III-d
		IV	IV	IV
X	5	V	–	V-a
		VI		V-b
XI	6	VII		V-c
XII	7	VIII		VI-a
XIII	8			VI-b
		IX		VII
XIV	9			
XV				
–	–	X		VIII

The chronology of Unit VI is constrained by a luminescence (pIR-IRSL) age of 198 ± 11 thousand years ago (ka) (1σ) obtained on optically-bleached potassium feldspar grains^50^. Two overlying units corresponding to calcite flowstones, one at the top of the sequence (Unit I) and one in the middle of the sequence (Unit IV) have been U–Th dated^50^. A series of U–Th samples obtained for the stalagmite of Unit IV produced a mean age of 100.3 ± 6.1 ka, placing this unit confidently within MIS 5c, while Unit I probably corresponds to MIS 2, between ~ 14 and 16 ka^50,51^. The presence of *Hystrix* sp. and *Iberomys cabrerae* in Unit III (subunit III-b) indicates a biochronological age for this unit of between ~ 90 and 60 ka BP^52^. Radiocarbon ages on animal bone remains, some of which were modified by humans, indicates that the human presence in Unit III spans > 51,000–44,210 cal BP^49^. This chronology corresponds to MIS 3, a period characterized by extreme and short-duration climatic oscillations^53^. Palaeoecological data place the initial formation of Teixoneres Unit III within a cold and humid period, which changes progressively to warmer periods with temperate and humid conditions, and finally turns cold and arid at the top of the unit^11,32,52^.

**Figure 3:**
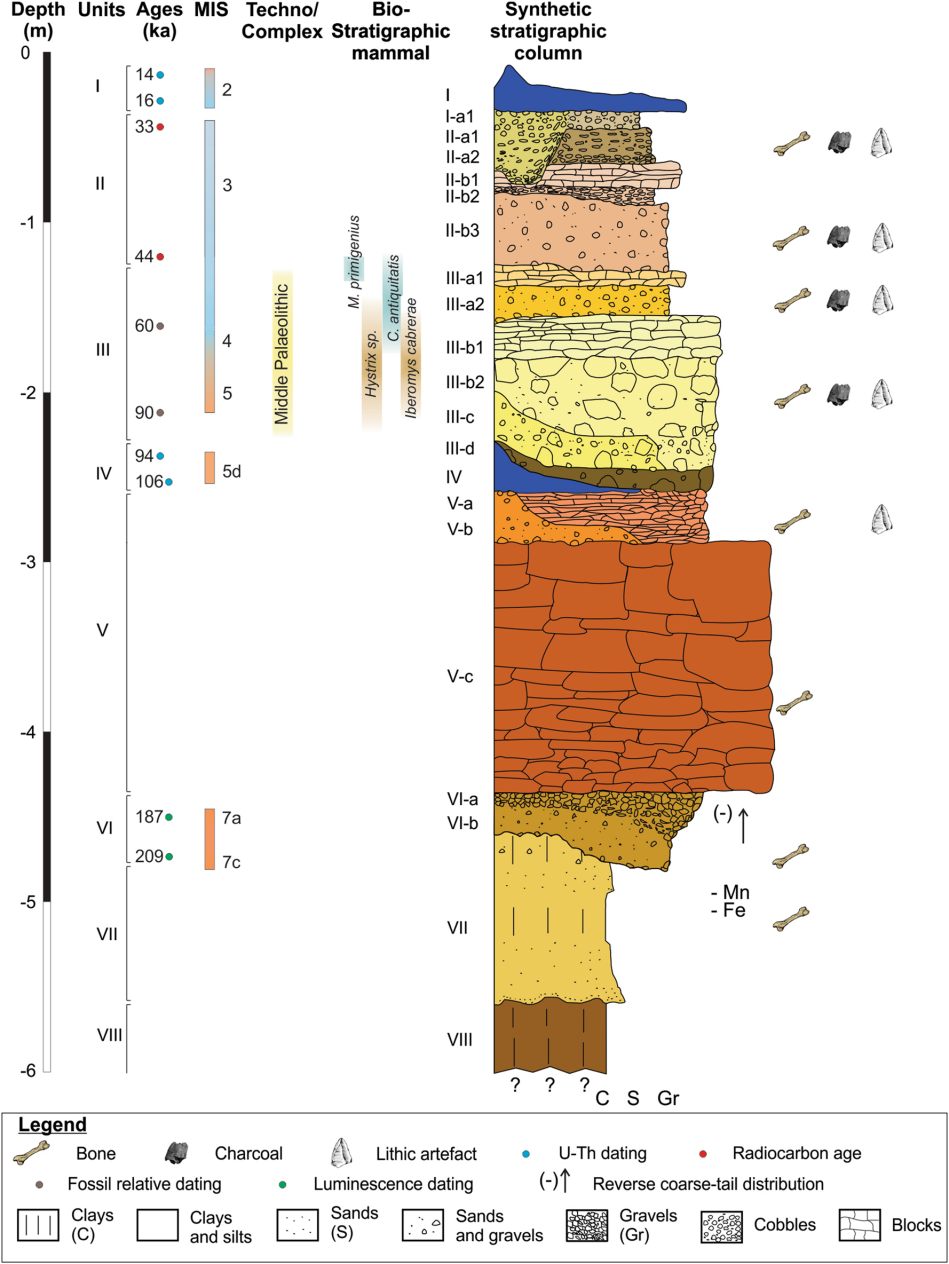
Synthetic stratigraphic column of Chamber X in Teixoneres Cave. References^29,32,49–52^.

Our latest analysis of the stratigraphic profiles (Supplementary Sect. 1) has permitted us to establish an improved stratigraphic correlation between the preserved units. This has allowed the development of a synthetic stratigraphic column, which comprises the different units and subunits that form the combined Teixoneres deposit (Fig. 3).

## Materials and methods

The archaeostratigraphic and spatial analyses presented here were conducted on the outer area and southern part of the inner area of Chamber X (Fig. 2d). Chamber X has a total surface area of approximately 93 m^2^, which can be sub-divided into the inner (62 m^2^, including the unexcavated area and pits from 1950s and 1970s) and outer (31 m^2^) areas. The area considered for this study is around 71 m^2^.

During excavations, all items larger than 1 cm (bone specimens, lithic artefacts, charcoal fragments, and teeth) and other archaeological features (hearths) were recorded and registered following a Cartesian-coordinates system. This method ensures that the position of materials have been recorded as they are found at the site and enables reliable assessment of their relationships with other items. Limestone blocks larger than 10 cm have been registered with a central point taken at their base. All excavated sediment has been sieved (5 mm and 1 mm mesh size) to recover small elements and fragments.

Our latest description of the lithostratigraphic sequence of Teixoneres is based on observations and analysis conducted during the 2019 excavation season (see Supplementary Sect. 1). The archaeological data used to perform spatial analysis of Unit III were recovered during excavations undertaken between 2003 and 2017. The total sample size analysed includes 38,244 archaeopalaeontological materials and 5888 limestone blocks. The back part of Chamber X in the interior of the cave (Fig. 2d), which was used more frequently by carnivores as a den or refuge^28^, is not included in the spatial analysis as it is not possible to make a distinction between the different subunits of the stratigraphic sequence in this sector of the cave^49^.

In order to recognise stratigraphic variations in Unit III, three-dimensional projections of the archaeological record and limestone blocks were carried out. The development of vertical and horizontal spatial distributions of remains was performed using the software ArcGIS 10.5. To explore the concentration, dispersion or random nature of the distribution of materials, the Ripley's K function and the Average Nearest Neighbour (ANN) application has been carried out^26,54–58^. Kernel density analysis has been applied in order to identify zones of accumulation of materials and classification of lower and higher density areas. This method calculates the density of point features around each raster cell, generating a smoothed map based on the location of points relative to one another^59–61^. We have applied a cell size of 1 cm and we tested several search radii, selecting a final radius of 0.50 m. The ArcGIS ‘mean center’ tool has been used for the purpose of measuring the centre of concentration of groups of remains. The mean centre is the average x and y coordinates of the group of selected objects in the study area. The combination of these analyses has allowed us to refine the initial spatial analysis of Unit III and their subunits, as well as undertake improved identification and location of the main areas of accumulation.

The spatial study incorporates data from lithic artefacts analyses, which provide basic information about the human presence in the cave and their occupational patterns. For this purpose, we have analysed raw material, lithic refits and size. This kind of study is useful for evaluating the dynamics and formation processes of the assemblages, evaluating the role of post-depositional processes, and obtaining more precise temporal data on the archaeological palimpsests^5,6,10,37,39,62,63^. All bone and dental remains have been analysed for anatomical and taxonomical identifications, as well as for palaeontological and taphonomic studies. Bone-induced modifications were analysed at both macroscopic and microscopic levels^64–66^. Bone specimens with burning damage were classified according to their structure, homogeneity and colour changes, which reflect different responses of the organic and inorganic components of the bones to increases in temperature exposure. We differentiate six general categories of intensity^67^, with degree 0 representing the unburned bones and degree 5 representing calcined bones. The intermediate degrees are 1, when the bone shows brown stains; degree 2, when it bears a homogeneous brown shade; degree 3, black; and degree 4, when the bone reaches grey schemes, sometimes with streaks of blue tones. The presence of bones with double colourations was also recorded. This combination of colouration is due to the fact that the entire surface was not exposed to fire with the same intensity^68^. Following Vaquero and Pastó^16^, the lithic and bone remains have been classified into three groups, according to their length and width: small (< 400 mm^2^), medium (between 400 and 1300 mm^2^) and large (> 1300 mm^2^).

## Results

### Definition of subunits in Unit III

**Figure 4:**
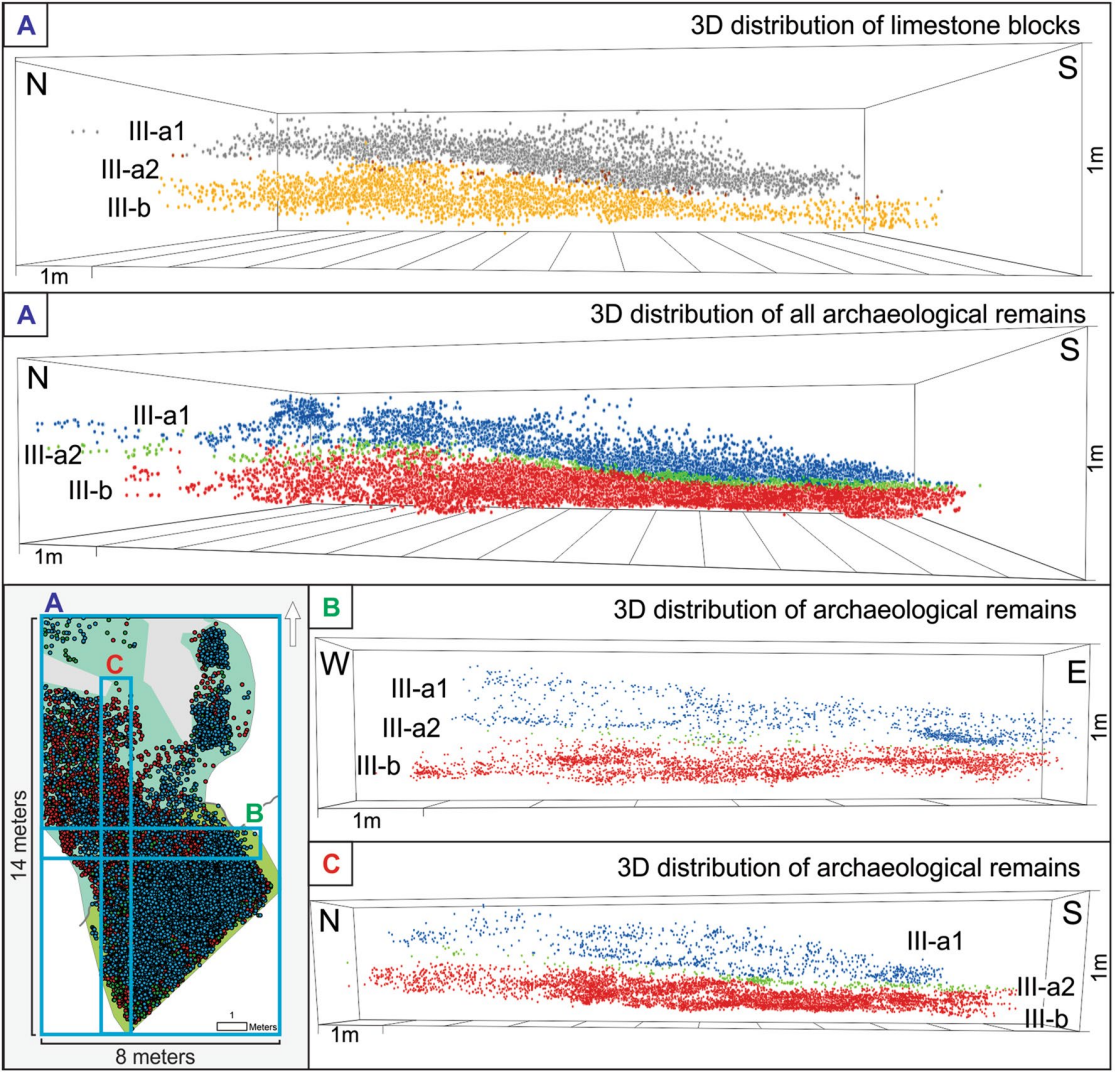
Vertical distribution of limestone blocks and archaeological items in subunits III-a1, III-a2 and III-b of Teixoneres Cave. The figure was prepared with the ArcGIS Version 10.5 software.

In previous studies, the lithostratigraphic sequence of Unit III at Teixoneres cave was subdivided into two subunits, III-a and III-b, following petrographic criteria, the disposition of the larger limestone blocks and general changes in the colour of the sediments identified during fieldwork seasons^28,49^. However, the three-dimensional projection of the limestone blocks in Unit III has allowed us to clearly recognise that subunit III-a can be further divided into two geologically-defined stratigraphic subunits, which have been named III-a1 and III-a2 (Fig. 4).

**Figure 5:**
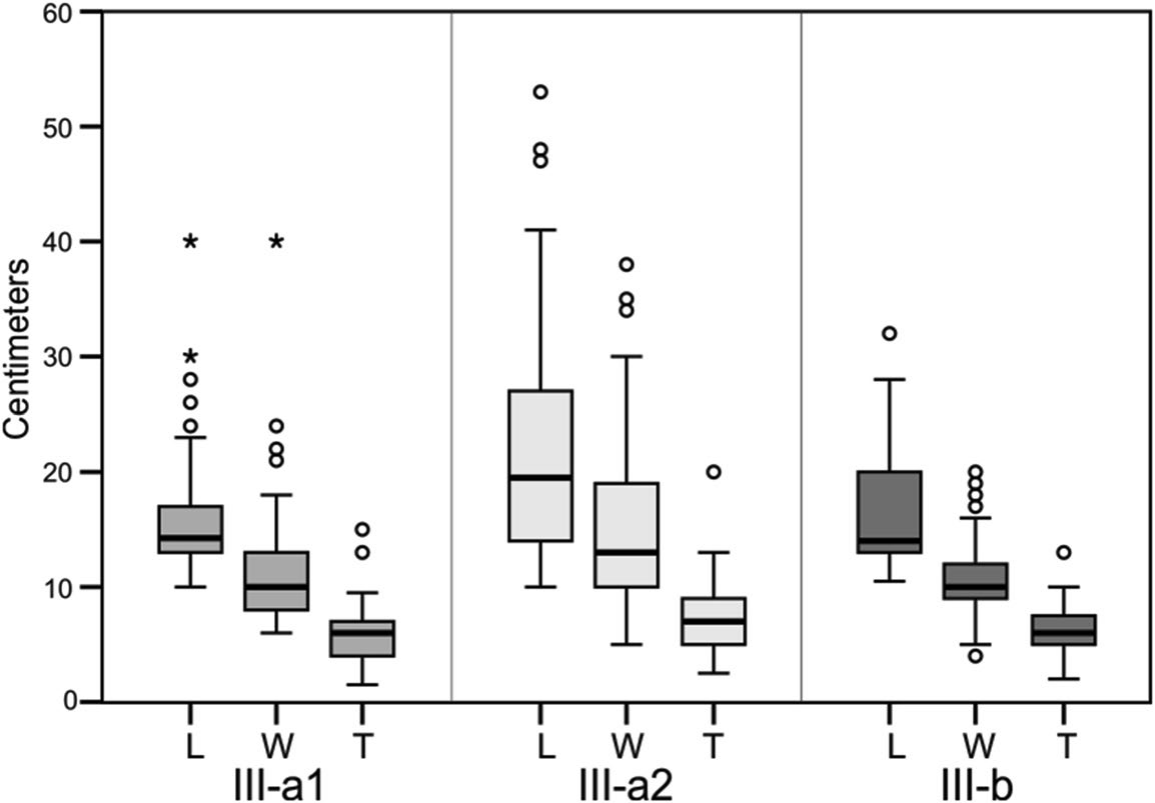
Boxplot of dimensions of the limestone blocks in subunits III-a1, III-a2 and III-b. *L* length, *W* width, *T* thickness.

The vertical projection and analysis shows that the upper part of subunit III-a is represented by a layer of sediments with the presence of limestone blocks along almost all its surface, albeit with a higher density of materials in the inner area. The latter is explained because the limestone blocks come from landslides that were generated from the roof and walls inside the cave. This subunit has been defined as III-a1 (Fig. 4). Underneath this subunit there is a thin layer of sediment, approximately 5–25 cm thick, which thins in a north–south direction in Chamber X, and is characterized by the almost total absence of limestone blocks. This subunit, called III-a2, corresponds to a period during which there was no detachments of limestone blocks in the cave, unlike subunits III-a1 and III-b. From the 5888 limestone blocks registered in Unit III, 3269 (55.8%) are in subunit III-a1, 58 (1%) are in subunit III-a2, and 2561 (43.2%) are in subunit III-b. The average limestone blocks length for subunit III-a1 is 17 cm (Std Dev 7.6 cm), compared to 22.3 cm (Std Dev 10.6 cm) for III-a2 and 18.7 cm (Std Dev 8.9 cm) for III-b. Figure 5 shows a boxplot with the dimensions of the limestone blocks from Unit III.

Although in the central sector of Chamber X (Supplementary Fig. S1) subunit III-b has been differentiated into two stratigraphic layers (III-b1 and III-b2), the high content of limestone blocks in the two subunits does not allow us to follow the same criteria of separation in the spatial analysis. As such, in the present study we have opted to analyse these remains as a single group, referred to as III-b. Future work will focus on higher resolution spatial analysis to verify whether this division is confined to the local geological level or whether it extends over a large area.

After recognising and defining the stratigraphic differences in Unit III, we have classified the recovered archaeological materials according to the three subunits (III-a1, III-a2 and III-b), taking into account the relief of each subunit. The three geologically defined stratigraphic subunits were then used as a minimum unit for horizontal intra-site spatial analysis. Table 2 shows the frequency of archaeological items identified in each of the stratigraphic subunits (Fig. 4). The volume of sediment excavated for each subunit was: 14.05 m^3^ (III-a1), 9.02 m^3^ (III-a2), and 13.19 m^3^ (III-b).

**Table 2:** Number of archaeological items from subunits III-a1, III-a2 and III-b.

Archaeological items	Subunit					
	III-a1		III-a2		III-b	
	NR	NR (m^3^)	NR	NR (m^3^)	NR	NR (m^3^)
Bones	7464 (82.7%)	314.5	1414 (78.2%)	71.9	23,091 (84.2%)	1796.9
Lithic artefacts	914 (10.1%)	38.5	261 (14.4%)	13.2	2682 (9.8%)	208.7
Teeth	567 (6.3%)	23.8	130 (7.2%)	6.6	1498 (5.4%)	116.5
Charcoal	76 (0.9%)	3.2	3 (0.2%)	0.1	144 (0.5%)	11.2
Total	9021 (100%)	380.1	1808 (100%)	92	27,415 (100%)	2133.4

*NR* number of remains.

Lithic raw materials with the highest frequency in Unit III correspond to artefacts manufactured from quartz and chert, which greatly exceed the rest of the identified rock types (Table 3).

**Table 3:** Frequency of lithic raw materials in subunits III-a1, III-a2 and III-b.

Lithic raw materials	Subunit		
	III-a1	III-a2	III-b
Quartz	611 (66.8%)	139 (53.2%)	1491 (55.6%)
Chert	217 (23.7%)	81 (31%)	937 (34.9%)
Quartzite	8 (0.9%)	5 (1.9%)	39 (1.5%)
Limestone	22 (2.4%)	8 (3%)	23 (0.9%)
Sandstone	5 (0.5%)	1 (0.4%)	31 (1.2%)
Hornfels	2 (0.2%)	1 (0.4%)	6 (0.2%)
Slate	4 (0.4%)	0	5 (0.2%)
Porphyry	0	0	1 (0.03%)
Other rocks	45 (4.9%)	26 (10%)	149 (5.5%)
Total	914 (100%)	261 (100%)	2682 (100%)

The frequencies of burned bones are 1170 (15.7%) for subunit III-a1, 209 (14.8%) for III-a2 and 2076 (9%) for III-b. Grade 3 is the most abundant modification type (1181 remains). A similar frequency of bones exhibit double colouration (1164 remains) (Table 4).

**Table 4:** Number of burned bone remains according to grades of colour modification.

Subunit	Grade					Double colour	Total	% Burned
	1	2	3	4	5			
III-a1	16	214	426	81	97	336	1170	15.7
III-a2	8	42	92	13	10	44	209	14.8
III-b	58	426	663	55	54	784	2076	9

The frequency of bone specimens with anthropogenic marks identified for subunit III-a1 is 100 (1.3%), compared to 16 (1.1%) for III-a2 and 132 (0.5%) for subunit III-b. Cut marks, chop marks, sawing marks and scrapes were recognised on bones. Of the total number of bones with anthropogenic marks in subunit III-a1, 53 are of small size, 28 of medium size and 19 correspond to large size remains. For subunit III-a2, five bones are of small size, four of medium size and seven of large size. Finally, for subunit III-b, 29 bones are of small size, 47 of medium size and 56 correspond to large size remains.

Additionally, the frequency of skeletal remains with marks generated by carnivores is 399 (5.3%) in subunit III-a1, 44 (3.1%) in III-a2 and 154 (0.6%) in III-b. Bones with crenulated edges, pits, digestion marks, furrowing, punctures and scores were identified. Of the total number of bones with carnivorous tooth marks in subunit III-a1, 213 are of small size, 140 of medium size and 46 correspond to large size remains. For subunit III-a2, 11 bones are of small size, 16 of medium size and 17 of large size. Finally, for subunit III-b, 40 bones are of small size, 61 of medium size and 53 correspond to large size remains.

The number of bone specimens and teeth identified in each subunit are presented in Table 5. Equidae and Cervidae are the most represented ungulates, while among the carnivores, the most represented species is *Ursus spelaeus*.

**Table 5:** Number of Specimens (NSP; including anatomic and taxonomically identifiable bone fragments, as well as fragments that were not attributed to a body-size class) and teeth by subunits III-a1, III-a2 and III-b at Teixoneres Cave.

Species	Subunit						Total
	III-a1		III-a2		III-b		
	Bone	Tooth	Bone	Tooth	Bone	Tooth	
*Ursus spelaeus*	16	35	8	13	8	12	92
*Canis lupus*	–	–	–	–	2	–	2
*Vulpes vulpes*	–	–	–	–	–	1	1
*Lynx* sp.	8	–	–	–	8	1	17
*Crocuta crocuta*	1	8	–	5	2	3	19
*Meles meles*	–	4	–	–	–		4
Unidentified Carnivora	6	9	–	2	23	15	55
*Mammuthus primigenius*	–	1	–	–	–	–	1
*Coelodonta antiquitatis*	–	7	–	2	–	5	14
*Equus* sp.	23	137	4	25	31	158	378
Unidentified Bovidae	10	9	1	–	14	11	45
*Bos/Bison*	2	3	2	5	13	15	40
Unidentified Caprinae	–	3	–	–	4	5	12
*Capra pyrenaica*	1	–	–	1	5	5	12
*Rupicapra pyrenaica*	2	1	1	1	1	–	6
Unidentified Cervidae	40	34	7	8	144	254	487
*Cervus elaphus*	59	53	9	9	222	225	577
*Capreolus capreolus*	7	3	6	1	45	23	85
*Sus scrofa*	–	4	–	–	–	3	7
*Castor* sp.	–	–	–	–	–	1	1
*Hystrix* sp.	1	6	–	–	2	5	14
*Oryctolagus cuniculus*	1	–	1	–	4	–	6
Unidentified Leporidae	159	11	19	–	290	9	488
Cheloniidae	10	–	–	–	3	–	13
Corvidae	1	–	–	–	–	–	1
Unidentified Birds	7	–	1	–	28	–	36
Large size	90	–	45	–	1032	–	1167
Medium size	624	–	278	–	4483	–	5385
Small size	306	–	181	–	5011	–	5498
Very small size	12	–	6	–	190	–	208
Unidentified bones	6078	–	845	–	11,526	–	1849
Unidentified teeth	–	239	–	58	–	747	1044
Total NSP	7464	567	1414	130	23,091	1494	34,164

### Distributional patterns in Unit III

#### Subunit III-a1

**Figure 6:**
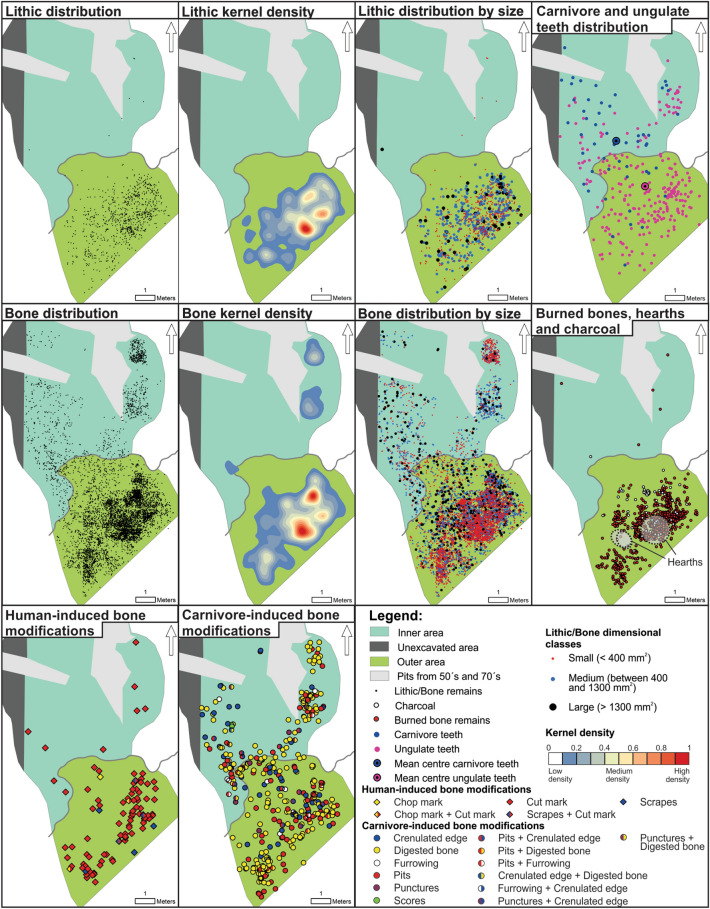
Spatial distribution of lithic artefacts, faunal specimens, burned bones, hearths, charcoal, carnivore/ungulate teeth, and human/carnivore-induce modifications on bones from subunit III-a1. The figure was prepared with the ArcGIS Version 10.5 software and the Adobe Photoshop CS5 Version 12.0.4 software.

The distribution of lithic artefacts in subunit III-a1 is almost entirely restricted to the outer area of the cave, where we observe three concentrations of high density of remains. Taking into account the dimensional classes of the lithic artefacts (large n = 57, medium n = 302, and small n = 555; Table 6), the refuse of small size are mainly in three high-density spots (Fig. 6). The knapping activities of quartz were mostly carried out on the central-eastern side of the outer area, whereas fewer artefacts were found in the western zone (Supplementary Fig. S4). Retouched tools and cores are distributed exclusively in the outer area (Supplementary Fig. S5, Tables S1 and S2). Chert stone tools, which are components of the toolkit and transported to the site already knapped, overlap in space with the quartz cores and by-products (Supplementary Figs. S4 and S5). A deeper examination of the distribution of cores and retouched tools reveals that a larger number of blanks are located at the periphery of the three high-density spots, in particular in the eastern area. The refitted artefacts (four refits comprising nine pieces) include a centripetal flake and a chert flake a *dos limité*, two fragments of a chert convergent tool, a centripetal core and a quartzite flake, and an exhausted Levallois recurrent centripetal core that, after a breakage, was reused for a short flaking production (Supplementary Fig. S6). In this latter example, it is worth noting that, after a knapping accident, the core was moved from the main area of activity, and transported a few meters to the eastern zone (Supplementary Fig. S6). After the second breakage, the core was discarded^45^. There are five connection lines. Four correspond to knapping activities and one to a fracture. Their distances vary from 30 cm to more than 2 m (Mean = 87.98 cm, Std Dev = 69.25 cm, Median = 80.36 cm) (Supplementary Table S3).

**Table 6:** Frequency and proportion of lithic artefacts and bone remains according to size categories and their location in Chamber X.

Archaeological items	Subunit	Small (< 400 mm^2^)		Medium (between 400 and1300 mm^2^)		Large (> 1300 mm^2^)		Total
		Inner area	Outer area	Inner area	Outer area	Inner area	Outer area	
Lithic artefacts	III-a1	6 (0.6%)	549 (60%)	0 (0%)	302 (33%)	1 (0.1%)	56 (6.1%)	914 (100%)
	III-a2	2 (0.7%)	122 (46.7%)	0 (0%)	107 (41%)	1 (0.4%)	29 (11.1%)	261 (100%)
	III-b	41 (1.5%)	1,511 (56.3%)	49 (1.8%)	897 (33.4%)	30 (1.1%)	154 (5.7%)	2682 (100%)
Bones	III-a1	659 (8.8%)	4226 (56.6%)	541 (7.2%)	1517 (20.3%)	137 (1.8%)	384 (5.1%)	7464 (100%)
	III-a2	20 (1.4%)	718 (50.8%)	87 (6.1%)	429 (30.3%)	61 (4.3%)	99 (7%)	1414 (100%)
	III-b	1157 (5%)	12,464 (54%)	1194 (5.2%)	6634 (28.7%)	469 (2%)	1173 (5.1%)	23,091 (100%)

Bone specimens are scattered across most of the excavated surface, although the highest density is located in the outer area of the cave. Large (n = 521) and medium (n = 2058) bones have been recorded in almost the entire analysed space, while those of small size (n = 4885) are clearly concentrated in the outer area (Fig. 6 and Table 6). When the spatial distribution of burnt bones, charcoal fragments and hearths is projected, it is observed that these are located almost exclusively in the outer area of the site (Fig. 6). Only in subunit III-a1 we have been able to identify three hearths, two of which are practically overlapping. These are located in the central area of the main entrance of Chamber X and associated with lithic artefacts and faunal fragments with anthropogenic marks. All of the identified hearths at Teixoneres are flat and seem to correspond to short-term combustion structures. These fireplaces are recognised mainly by the presence of a layer of red sediment covered by darker sediments with some charcoals and burned bones^11^. The spatial analysis of bones bearing anthropogenic damage shows a greater frequency in the outer area. The distribution of burned bones, according to burning damage degrees, presents a clear spatial pattern. There is a spatial trend in which the bones with the highest degrees of thermal alteration present a highly concentrated distribution, while the bones displaying lower degrees of burning have a wider spatial distribution (Supplementary Fig. S7). Taking into account a central point from the main hearth identified in subunit III-a1, it can be seen that distance-decay curves of the distribution of lithics, bones, burned bones and charcoal fragments exhibit a high concentration in the space occupied by this feature and its frequency decreases as the distance from the hearth increases (Supplementary Fig. S8). Cut marks are the most represented in the space, and chop marks and scrapes have low frequencies. The average size (length) of bones with anthropogenic marks is 40 mm. Bone specimens with carnivore modifications are distributed both in the inner and in the outer area of the cave. Crenulated edges, pits, digestion, furrowing, punctures and scores were recognised. Carnivore teeth are spatially distributed mainly in the inner area of Chamber X, while ungulate teeth are present in higher frequencies in the outer area of the cave. The mean centre of both distributions confirm this spatial trend (Table 5 and Fig. 6).

#### Subunit III-a2

**Figure 7:**
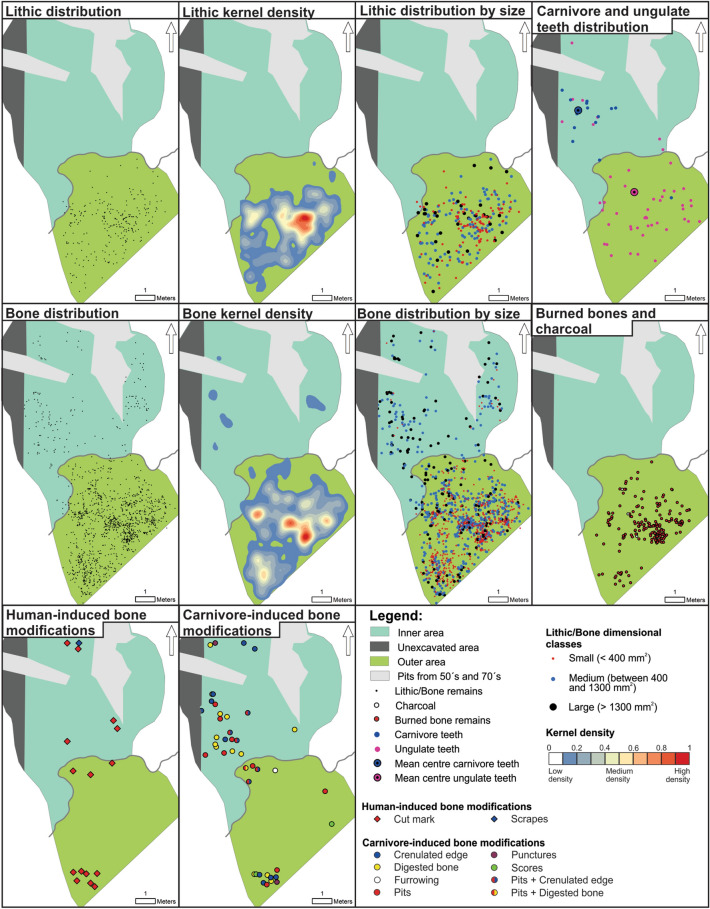
Spatial distribution of lithic artefacts, faunal specimens, burned bones, charcoal, carnivore/ungulate teeth, and human/carnivore-induce modifications on bones from subunit III-a2.

In subunit III-a2, the number of lithic artefacts is small and they are distributed in the outer area of the cave, as observed in subunit III-a1. In this subunit, small-sized pieces (n = 124) predominate over medium (n = 107) and large-sized artefacts (n = 30) (Table 6). Small size lithic artefacts show a high density spot located in the centre of the entrance of Chamber X, and a smaller one close to the western side of the drip-line (Fig. 7). Quartz and chert artefacts show a similar distributional pattern, although the latter are more concentrated (Supplementary Fig. S4). Refitted chert and metamorphic rock artefacts are absent from the assemblage, suggesting that cores and stone tools transported to the cave as components of the toolkit were discarded without further reduction or reshaping activities. The spatial distribution of the cores and retouched tools indicate that a larger number of items are located in the western side of the outer area and in a peripheral zone in comparison with the main high-density spot (Supplementary Fig. S5, Tables S1 and S2).

Bone remains are distributed throughout the analysed space, except in the case of burned bones and charcoal fragments, which are mainly concentrated in the outer area. Bones with burning degree 5 are highly concentrated, while the lower grades are found distributed throughout the outer area (Supplementary Fig. S7). When considering the dimensional classes of bones, it can be seen that those of small size (n = 738) are located exclusively in the outer area, while those of large (n = 160) and medium size (n = 516) are distributed both in the entrance and in the inner part of the cave (Fig. 7 and Table 6). The frequency of bones with anthropogenic modifications is low, but cut marks, incisions and scraping marks were recognised. These types of marks are distributed throughout the analysed area, as are the bones showing carnivore-induced modifications. The latter show crenulated edges, pits, punctures, furrowing and digested bones were identified (Fig. 7). The average size (length) of bones with anthropogenic marks is 66 mm. The southern sector of the outer area also preserves a small concentration of specimens with anthropogenic marks and others with signs of action by carnivores. The distributions of ungulate and carnivore teeth show a different spatial pattern for both groups of animals. Ungulate teeth are located mainly at the entrance of the cave, while carnivores are restricted to the inner area, as confirmed by the mean centre of the distributions (Table 5 and Fig. 7).

#### Subunit III-b

**Figure 8:**
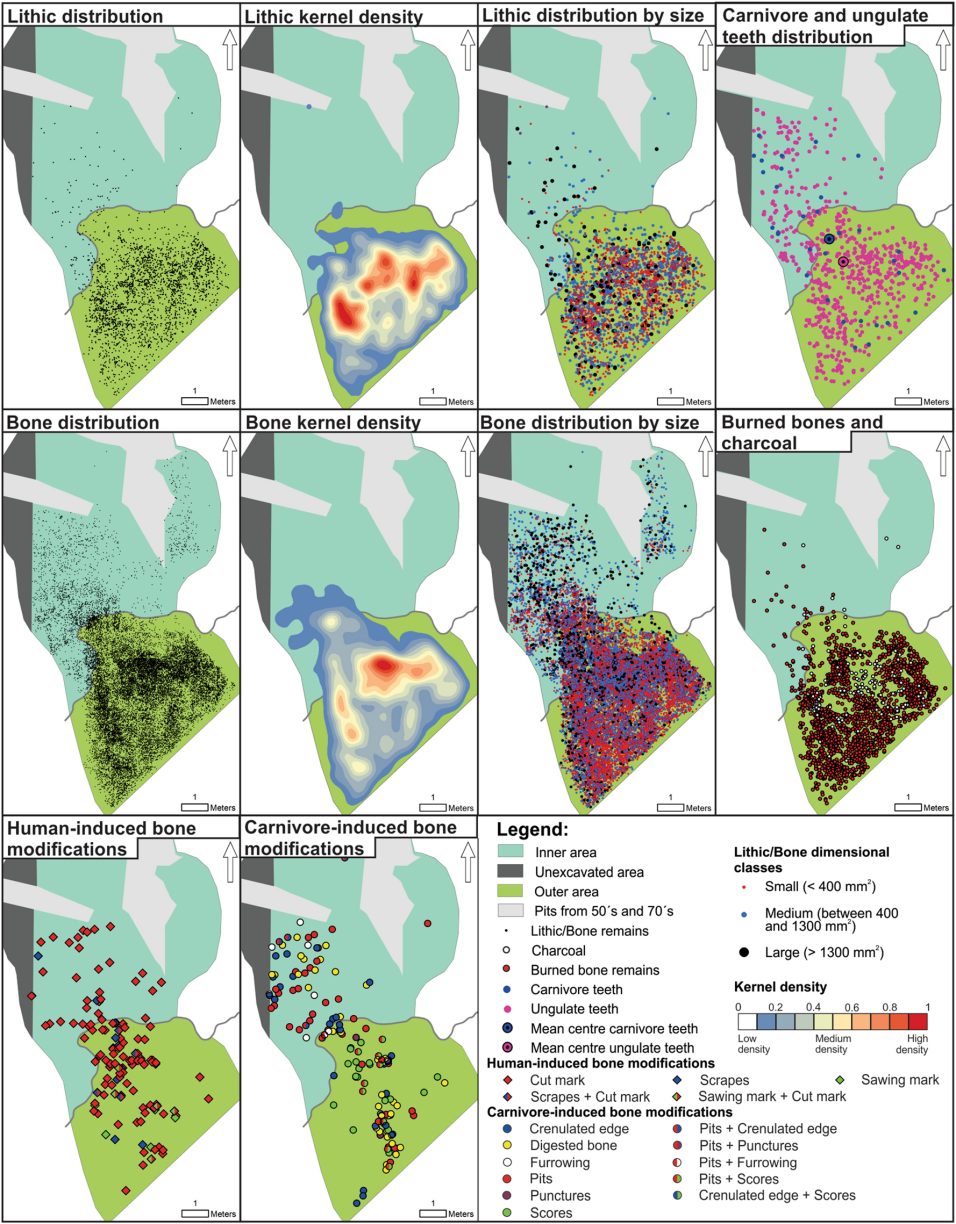
Spatial distribution of lithic artefacts, faunal specimens, burned bones, charcoal, carnivore/ungulate teeth, and human/carnivore-induce modifications on bones from subunit III-b.

The lithic materials from subunit III-b show a high degree of scattering across the whole surface at the entrance of the cave. However, by differentiating this assemblage according to dimensional classes, it can be observed that the smallest fragments (n = 1552) are located in the outer area, and are concentrated in well-delimited accumulations in some sectors. Medium (n = 946) and large size artefacts (n = 184) are not only scattered at the entrance of the cave but also in the inner area (Fig. 8 and Table 6). Artefacts in quartz and chert have a broad and similar distribution throughout the outer area of the cave (Supplementary Fig. S4). Cores and retouched tools are mostly located close to the drip-line and few stone tools are found in the inner area (Supplementary Fig. S5, Tables S1 and S2). The refitted artefacts (four refits comprising eight pieces) include two cortical core–edge chert flakes, a flake prepared with core convexity and a unidirectional chert flake, a flake prepared with striking platform and a chert fragment, and an unidirectional core, and a quartz flake (Supplementary Fig. S6). The high frequency of chert artefacts and the few refits suggest that cores and stone tools were produced elsewhere in the landscape, and transported to the site already configured^45^. The four connection lines correspond to conjoining of knapping activity by-products, and are located in the western side of the accumulation. The distances of the connection lines vary from 1 m to more than 3 m (Mean = 181.7 cm, Std Dev = 93.1 cm, Median = 150.29 cm) (Supplementary Table S3).

Bone fragments are distributed across the excavated surface, with the exception of burned bones, which are concentrated in the outer area. This pattern coincides with the spatial distribution of charcoal, as can be observed in Fig. 8. Numerous spots displaying high frequency of bones with burning degree 5 covered much of the cave’s entrance (Supplementary Fig. S7). Small sized bones (n = 13,621) are located mainly in the outer area of the cave, while large (n = 1642) and medium sized bones (n = 7828) show a wider distribution (Fig. 8 and Table 6). The distribution of bone specimens with anthropogenic modifications and those with carnivore damage is similar across the surface excavated in subunit III-b. Incisions, scrapes and sawing marks were identified among human modifications. Crenulated edges, pits, furrowing, punctures, scores and digested bones are the most significant modifications associated with carnivore activities. The average size (length) of bones with anthropogenic marks is 65 mm. Ungulate and carnivore teeth are spatially distributed throughout the entire analysed space, with the ungulate teeth showing a wider distribution in the outer area of the cave (Table 5 and Fig. 8).

The distribution pattern for subunits III-a1, III-a2 and III-b has been analysed by applying different statistical tests, such as Average Nearest Neighbour (ANN) and Ripley’s K Function (Table 7). The K-function shows that the empirical (observed) curve is higher than the theoretical (expected) curve, indicating the presence of more neighbours than we would expect if the pattern was random (Supplementary Fig. S9). This positive association is consistent with a concentrated pattern, something that has been verified by combining this test with ANN. The statistical analyses performed point to a less than 1% probability that this pattern could be due to random chance.

**Table 7:** Statistical results obtained from the application of ANN and Ripley’s K Function.

	Subunits		
	III-a1	III-a2	III-b
**Average nearest neighbour (ANN)**			
Observed mean distance	2.65	6.42	1.78
Expected mean distance	4.57	11.52	2.87
Nearest neighbour ratio	0.58	0.55	0.62
z-score	− 89.22	− 36.57	− 125.66
p-value	0	0	0
**Ripley's K function**			
Maximum difference observed-expected	110.54	118.15	109.88

## Discussion

The application of GIS tools for studying the spatial distribution of archaeological items from Unit III at Teixoneres Cave has allowed us to clearly recognise three geologically-defined stratigraphic subunits and isolate the materials distributed in each one of them, taking into account the relief of the ground for their analysis. By examining the vertical distribution of all archaeological items and limestone blocks it has been possible to observe a stratification that was possibly not significantly affected by processes of vertical material mobilization. This observation is important because carnivore activities have been constant throughout the formation of Unit III, and some of these predators could have undertaken fossorial activities within the burrows they created for different purposes^69^. However, the clear stratification of Unit III allows us to postulate that the activity of carnivores is unlikely to have been intense, in the sense that they were relatively unimportant agents of vertical mobilization of materials in the southern sector of Chamber X.

The horizontal spatial distribution analysis performed for Unit III indicates an occupational pattern that is repeated in all of the studied stratigraphic subunits. This pattern consists of fire-related areas located at the entrance of the cave and the development of multiple activities, such as knapping quartz pebbles, resharpening of chert tools and processing of ungulate carcasses (mainly horses and red deer). Such activities generated well-defined spatial concentrations of remains in the outer area of the cave, where lithic artefacts, faunal specimens, charcoal fragments, fireplaces and burned bones are clearly dominant. The accumulation of charcoals and thermally altered bones, especially those that show high degrees of burning damage, in well-delimited zones seem to indicate that hearths were placed in specific areas, even though the structural features of these were not preserved in the archaeological record, as seen for subunits III-a2 and III-b. In this sense, some sedimentary contexts do not exhibit the kind of potential preservation that is required for evidence of fire to survive, so spatial analysis of distributional use patterns is a good tool to infer the potential presence of hearths that are no longer visible (e.g.^26,70–74^). The features of the hearths identified in subunit III-a1, such as flat structures together with their dimensions and lack of specific preparation, suggest that these structures are the result of repeated short-term human occupations. The distribution of the archaeological items shows that the fireplaces were considered referential axes around which the space was organized, with most of the human activities concentrating around them.

The spatial distribution of materials is similar in the three subunits. However, in subunit III-b there is a higher density of remains, which could suggest that the cave was visited by Neanderthals more assiduously or intensely than in the upper subunits. Studies of tooth microwear patterns for subunit III-a^31^, combined with an estimation of the seasonality through dental eruption of the ungulates, suggest repeated seasonal occupations of the site at all seasons^31^. However, for subunit III-b, tooth microwear, tooth eruption patterns, and cementochronology^31,75^ indicate occupation patterns characterised by repeated short settlements occurring in different seasons (spring, summer and winter). These seasonality studies in Teixoneres reinforce the idea of a succession of short-term occupations at different times of the year.

During these recurrent visits at the site, Neanderthals transported toolkits composed of heterogeneous artefacts (large flakes, retouched artefacts and cores) in chert and other rocks (metamorphic, sedimentary and igneous), and only the local quartz pebbles were knapped completely on site for providing more fresh cutting-edges^45,46^. Although the distribution of the lithic materials indicates a recurrent spatial patterning in the outer area of the cave for all subunits, the Kernel density mapping reveals some slight differences. These spatial variations include the location of the high and medium density spots that diachronically changed from the area closer to the drip-line in subunit III-b, to the central-western zone in subunit III-a2, and to the central-eastern area in subunit III-a1. All these main accumulations are located near fire use related zones and to some extent correspond to high Kernel density bone distributions. In subunit III-b, the comparison between scattered quartz and chert artefacts indicates a greater overlap between the local knapping production and the discard of the transported toolkit, whereas in subunits III-a2 and III-a1 some distributional variations are documented. These latter differences are also recognised in the spatial patterning of cores and retouched tools. From a broader perspective, the Neanderthal occupational dynamics at Teixoneres Cave were diachronically recurrent in the outer cave area, whereas the density of lithic artefacts is considerably lower towards the inner area.

The number of identified lithic refits is small and comprises mostly chert artefacts. The large amount of conjoining blanks in the assemblage contain two reffiting elements, with only in one example comprising three refitted pieces. The technical categories of the refitting material include primarily knapping by-products, while two are retouched tools, and five are cores and core fragments. These artefacts were produced using Levallois strategies. The low number of lithic refits is expected if we consider that, except for quartz, the other raw materials were transported to the site already knapped and configured. The distances of the connection lines are within the normal dispersion range observed by experimental archaeology for on-site knapping sequences; that is, generally less than 1 m in diameter and rarely exceeding 2 m^76–79^. However, three connections exceed the normal dispersion range suggesting intentional anthropogenic displacements from the main knapping areas to peripheral zones. Natural processes (e.g., slope and erosion) cannot have produced these movements, and the transport from one sector to another shows intra-site intentional mobility of pieces in the frame of Neanderthal daily activities.

Charcoal fragments are concentrated within small accumulations located in the outer area of the cave. These remains are of small size and low weight, which makes them highly susceptible to transportation by different agents. When charcoal fragments lay on the surface for long periods, these can disappear due to long-term exposure to wind, rain, animal activities, trampling and other environmental conditions^70^. The small accumulations of charcoal fragments observed in Unit III could be related to the low incidence of action by wind, water or other agents that could have dragged these remains to small cavities or irregular zones on the surface.

A large percentage of bone specimens show signs of burning, which could be the consequence of different processes. Thus, these remains could be grouped into three main categories.

1. Bones that were subjected to fire exposure before consumption (roasting).
2. Bones that were thrown into the fire for cleaning purposes after meat removal (waste elimination) or the use of bones as fuel.
3. Bones that were burned as a result of unintended or accidental processes, including post-depositional phenomena (e.g., alteration when fireplaces are set up on bones buried close to the surface).

On this basis, we consider that the high percentages of burning damage observed (grades 3 and 2) is the result of bones undergoing processes described in categories 1 and 2. Similarly, the presence of bones with double colourations, which likely results when the entire surface of the bone is not exposed to the same fire intensity, is also possibly due to roasting before defleshing^80^. This occurs because when prey are placed on the hearth for cooking, the portions of bone with minimal flesh covering receive more intense heat treatment, which results in variable burning effects and high colour tonalities^68,80^. Meanwhile, the portions of the bone that are covered by flesh maintain their original colour and, on occasions, acquire low tonalities (burning damage grades 1 and 2) in places with less muscle mass. In contrast, bones that exhibit grades 4 and 5 burning damage were either thrown onto the fire after consumption or were already deposited bones (with or without signs of thermal alteration) onto which subsequent hearths were made by hominins. Additionally, processes such as trampling or falling limestone blocks could increased the fragmentation of this type of remains. It is important to note that non-homogeneous colourations similar to those described by Bennett^81^ for bones burned after burial have also been identified, indicating clearly overlapped activities related to the use of fire at the site.

The spatial analysis of bones bearing anthropogenic and carnivore modifications shows a similar distribution at the site. These remains are distributed across the inner and outer area of the cave. The presence of carnivore-modified bones in the outer area of the cave supports the hypothesis of short-term human occupations. The repeated occupation and abandonment of the cave by human groups would have favoured the presence of carnivores at the site, attracted mainly by the animal remains left by humans^18,25,27^. The consumption of human refuse by carnivores can result in the fragmentation and even the disappearance of several bones and other anthropogenic traces such as hearths and lithic tools^18^. The continuous succession of short-term human occupations and carnivores in the cave, added to the low sedimentation rate, generated a palimpsest.

Carnivore teeth from subunits III-a1 and III-a2 are mainly distributed in the inner area of the site, suggesting that these teeth belong either to carnivores that occupied these inner areas as a dens^82^, or to carcasses that were accumulated by other non-human predators. The systematic use of carnivores as hominin prey is not commonly documented in Middle Palaeolithic archaeological sites. In some cases, evidence of this type of activity has been explained as a process of eliminating competitors in the environment or as occasional hunting events^27,83^. In subunit III-b carnivore teeth are scattered throughout the entire analysed space, while the ungulate teeth are spatially restricted to the outer area of the site, as seen in the other subunits. The introduction of ungulate carcasses to the cave would have occurred due to the action of hominins and carnivores. However, the distribution of ungulate teeth in the outer area is closely related with lithic, bones and hearths, indicating that the presence of this type of fauna could correspond to the action of the human groups. It is worth highlighting that, for all subunits, the spatial distribution of carnivore teeth does not resemble the spatial pattern represented by the lithic remains, charcoal fragments and bone specimens, which make up well-defined concentrations around fireplaces in the outer area of the cave. These different distribution patterns suggest the existence of different agents in the formation of the main materials accumulations and the relatively low incidence of natural post-depositional processes that could have removed, mixed or accumulated the remains (e.g., water flows). This evidence permits us to infer the relatively good stratigraphic integrity for subunits III-a1, III-a2 and III-b. Therefore, the highest density of remains left by humans at the entrance of the cave, in contrast to the distribution of carnivore teeth and bones with carnivore-induced modifications, supports the hypothesis of alternation between hominin and carnivore occupations of the cave, where carnivores seem to have preferred the inner sheltered zones.

Two cold-adapted mammal species, *Coelodonta antiquitatis* and *Mammuthus primigenius*, have been identified in Unit III at Teixoneres during MIS 3, a period characterized by extreme and relatively short-lived climatic oscillations^32^. Our analyses show that the 14 teeth remains of *Coelodonta antiquitatis* are present throughout the analysed sequence, while the sole tooth of *Mammuthus primigenius* was from subunit III-a1. Moreover, the highest percentage of remains (teeth) of these two species predominate in subunits III-a1 (1.4%) and III-a2 (1.5%), compared to subunit III-b (0.3%). Additionally, the association between these two species, which according to Alvarez Lao et al*.*^32^ is a clear indicator of cold and arid environments in the Iberian Peninsula, occurs in subunit III-a1. Therefore, the higher representation of two cold-adapted species in the upper subunits could be indicative of a higher evidence of cold and arid events during this period or an increment in the intensity of these cold events. As already mentioned, the archaeological evidence suggests that the cave was visited by humans less frequently in subunits III-a1 and III-a2 than in subunit III-b. This is an important point for future evaluation to determine whether the relatively lower human presence in subunits III-a1 and III-a2 was the result of a greater magnitude in severe climates and poorer environments, which would not have favoured human presence in the area.

The low sedimentation rate detected in Unit III (between 35 and 65 cm of deposit in ˃ 7000 years) favours the mixing of materials from different occupation events over time and the formation of palimpsests^11^. Human groups that repeatedly inhabited the site were able to reuse the remains left by previous groups and even make use of some of the leftover debris such as lithic raw materials, vegetables or faunal bones; a recycling behaviour that has been detected in other archaeological sites (e.g.^3,38,84,85^). Likewise, the visible presence of remains left by humans on the floor surface could have conditioned or structured the use and reuse of space inside the cave during subsequent occupations^3^.

The attractiveness of caves as natural refuges for both humans and carnivores could have generated a competition for space at different times in the past. Nevertheless, several authors agree that Neanderthals were superior to carnivores in the context of competitive relationships and the occupied space (e.g.^11,86^). Therefore, the presence of humans in the caves would have played an important role in the carnivore ethology regarding their stay at the cave: in the absence of humans, the place would have been used mainly as a den for bears, hyenas and other smaller carnivores or raptors, but the presence of human could have lead carnivores to act as marauders or opportunists in search of potentially consumable elements^27^. Once the cave was abandoned by hominins, the smells produced by the discarded remains likely attracted carnivores to the site^23,87–89^. Furthermore, the natural deaths of carnivores (and hominins) in caves could have attracted other scavengers to the site^11^. This dynamic use of the caves is likely to have generated some of the palimpsest characteristics of the archaeopaleontological deposit at Teixoneres. The patterns observed could be the consequence of alternate occupations of hominins and carnivores and, in both cases, these occupation periods would have been traversed by visits of marauding carnivores. In order to understand how the interaction between humans and carnivores developed, it is also necessary to appreciate that in some cases there was not only competition for certain resources but also for spaces with conditions that favoured habitability.

Similar to Teixoneres, many other Middle Palaeolithic cave sites across Europe display a succession of short-term human occupations with co-occurrences of large carnivores habitation^26,90–97^. At these sites, the alternation of remains left by both biological entities often causes problems when trying to understand the processes leading to assemblage formation, such as isolating specific episodes within the same archaeostratigraphic unit or determining intra-site anthropic spatial organization^18,19,86^. Rosell et al*.*^11^, p. 208, point out that Teixoneres was used in winter by bears during the hibernation season. The occupation seasons of hyenas and other predators is unknown. Meanwhile, human groups seem to have taken advantage of Teixoneres mainly during seasons in which the environment contained high ecological energy, which corresponds with the red deer during their mating season (autumn) and with the presence of migratory ungulates (summer).

Teixoneres Cave shows complexity in the occupation of the site by human groups and carnivores, which, in addition to low sedimentation rate and diverse natural processes, results in complex site formation processes. However, the corpus of information analysed in this article has allowed us to test and confirm the initial working hypothesis, which posits that the site was occupied by Neanderthal groups in a similar way (structured and consistent) through the entire temporal range covered by subunits III-a1, III-a2 and III- b, and that this favoured the creation of a spatial pattern dominated by fire use related zones in the outer area of the cave. At the same time the presence of carnivores is clearly represented in the archaeological record, although they did not generate significant spatial perturbations of the site.

## Conclusions

Caves are considered as refuges because they provide protection against some elements such as wind, rain, snow and sun, as well as predators and other humans. In many cases, caves can be found in the proximity of different types of resources, making them very attractive spaces for human occupation. Many of these same qualities also make caves attractive for occupation by carnivores. At the intra-site level, certain areas of the caves have suitable conditions for the performance of specific activities. This is considered to be the case for the outer cave area at Teixoneres, a sector in which lithic knapping and faunal processing activities around hearths were carried out by Neanderthal groups. This area is drier and better ventilated than the inner sector, thus allowing for improved ignition and maintenance of fireplaces, as well as being better illuminated by sunlight. In addition, the outer area is framed by the rock walls of the cave, which results in a limited floor surface that is approximately 6 m wide. The dimensions of this area thus also conditioned the use of the space inside the cave during the different and repeated periods of human occupation. In this way, cave sites such as Teixoneres exhibit a series of attributes that make them attractive spaces for habitation.

Through intra-site spatial analysis, together with a detailed study and characterization of the stratigraphy, it has been possible to reconstruct the settlement dynamics in relation to the human activities performed at Teixoneres Cave. The presence of fire use related zones reveals a specific spatial organization of the social activity around it, and provides evidence for a formal conceptualisation of domestic space. The study of the spatial organization patterns is an important issue to consider at cave sites, since it opens up the possibility to determine aspects of complex human behaviour occurring during the Middle Palaeolithic.

## Supplementary Information

Supplementary Information.
