# Supplementary material for: Examining Neanderthal and carnivore occupations of Teixoneres Cave (Moià, Barcelona, Spain) using archaeostratigraphic and intra-site spatial analysis
Source: Sci Rep. 2021 Feb 22;11:4339. doi: 10.1038/s41598-021-83741-9 (PMC7900232; doi:10.1038/s41598-021-83741-9)
Supplement: Supplementary file 1 — Supplementary Information. [file 41598_2021_83741_MOESM1_ESM.docx]

Examining Neanderthal and carnivore occupations of Teixoneres Cave (Moià, Barcelona, Spain) using archaeostratigraphic and intra-site spatial analysis

Leandro Zilio^1,^ *, Heidi Hammond^1^, Theodoros Karampaglidis^2,^ ^+^, Laura Sánchez- Romero^3,^ ^+^, Ruth Blasco^4,5,^ ^+^, Florent Rivals^4,5,6,^ ^+^, Anna Rufà^7,^ ^+^, Andrea Picin^8,^ ^+^, M. Gema Chacón^4,5,9,^ ^+^, Martina Demuro^10,^ ^+^, Lee J. Arnold^10,^ ^+^ and Jordi Rosell^4,5,^ ^+^

^1^ Consejo Nacional de Investigaciones Científicas y Técnicas (CONICET), Universidad Nacional de la Patagonia “San Juan Bosco”, Facultad de Humanidades y Cs. Sociales, Esquel, 9200, Argentina

^2^ MONREPOS, Archaeological Research Centre and Museum for Human Behavioural Evolution, Schloss Monrepos, Neuwied, 56567, Germany

^3^ Human Evolution Research Center, 3101 Valley Life Sciences Building, University of California, Berkeley, 94720, USA

^4^ Institut Català de Paleoecologia Humana i Evolució Social (IPHES-CERCA), Zona Educacional 4, Campus Sescelades URV (Edifici W3), 43007 Tarragona, Spain

^5^ Universitat Rovira i Virgili (URV), Departament d’Història i Història de l’Art, Tarragona, 43002, Spain

^6^ ICREA, Barcelona, 08010, Spain

^7^ Univ. Bordeaux, CNRS, MCC, PACEA, UMR 5199, F-33600 Pessac, France

^8^ Max Planck Institute for Evolutionary Anthropology, Department of Human Evolution, Deutscher Platz 6, 04103 Leipzig, Germany

^9^ UMR7194 Histoire naturelle de l'Homme préhistorique (HNHP), Museum National d'Histoire Naturelle (MNHN), CNRS, Université Perpignan Via Domitia, Alliance Sorbonne Université - Musée de l’Homme, Place du Trocadéro 17, 75016 Paris, France

^10^ School of Physical Sciences, Environment Institute, and Institute for Photonics and Advanced Sensing (IPAS), University of Adelaide, North Terrace Campus, Adelaide, 5005, Australia

* [leandrozilio@yahoo.com.ar](mailto:leandrozilio@yahoo.com.ar)

^+^ these authors contributed equally to this work

# SUPPLEMENTARY INFORMATION – section 1

**Stratigraphy of Teixoneres Cave**

The lithostratigraphic analysis was carried out using profiles perpendicular and parallel to the wall of the cave. The exposed stratigraphic profiles studied were the West profile and the East profile (Fig. 2d). The first of these profiles is located to the west of the central sector of Chamber X, and represents the same stratigraphic sequence described by Talamo *et al.*^1^. This profile is approximately 8 m long and 2 m high and contains Units I, II and III^1^. The second profile is located in an open pit from the 1950s and 1970s, located in the central-east sector of Chamber X. This profile is approximately 5.5 m long and 3 m high, and contains Units IV to VIII. These profiles were individually analysed and subjected to archaeostratigraphic criteria^2–6^, in such a way that it was possible to isolate and define the limits of units and subunits.

With the aim of obtaining a detailed description of the units, stratigraphic data have been combined with a preliminary spatial analysis performed on the whole deposit. At this point, it is important to highlight that the current study follows the basic units and nomenclature described in Talamo *et al.*^1^. Undertaking correlations with the older studies undertaken at Teixoneres is complicated, since the levels were only described at the local scale and part of the original stratigraphic sections no longer exist. In the present study, each layer has been described according to its colour, thickness, shape, particle size, roundness of the gravels, cobbles and blocks, texture matrix, cementation, autochthonous-allochthonous sediments, lithology of the gravels, cobbles and blocks (monomictic, oligomictic and polymictic) and archaeopalaeontological data. From the bottom to the top, we characterize the stratigraphic units and subunits defined for Chamber X as follows:

## Unit VIII

This unit is made of bright dark brown clays (80-90%) with high plasticity, and fine dark yellow sand with wispy laminations, discontinuous streaks and lenses (0% coarse - 100% fine). Its known estimated thickness

is approximately 60 cm. This unit is clearly eroded by Unit VII. Located in the central pit, current squares N/16-17 of the west stratigraphic profile (Figs. S1 and S2).

## Unit VII

This is an allochthonous unit of fluvial origin^7^, comprising clay lamination suspension deposits with depositional shear sorting. The unit contains fine, dark yellow, highly plastic clayey sands, and bright brown, high plasticity interlayer clays (20-30 cm). Its structure is stratified and clearly deformed by the fall of blocks from the cavity ceiling (0% coarse - 100% fine). Its known estimated thickness is approximately 70-80 cm. In the upper part of this unit, which is in contact with Unit VI, the presence of large mammals is recorded. Located in the central pit, current squares N/15-17 of the west stratigraphic profile (Figs. S1 and S2).


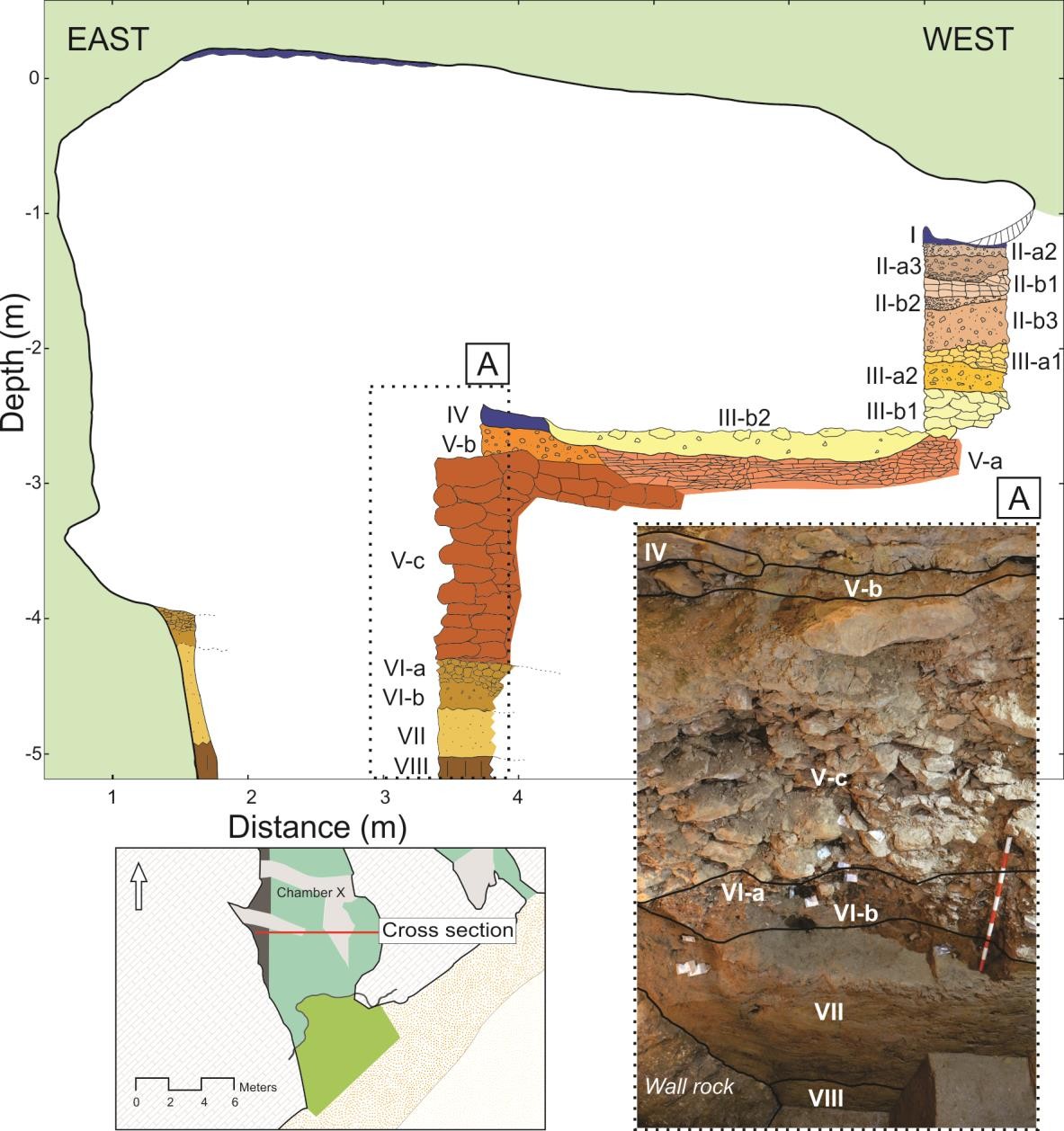


**Figure S1.** Stratigraphic sequence and East profile of Chamber X in Teixoneres Cave. Cross section made during 2019 fieldwork.

## Unit VI

Unit VI is separated into two subunits (Figs. S1 and S2):

- **Subunit VI-a**: Lenticular subunit with massive structure, moderately sorted with reverse coarse-tail distribution, comprised mostly of equant medium-sized subangular limestone clasts (its major axis between 6-14 cm) and well-graded middle gravels, and with loamy-clayey loose matrix of dark grayish-pale brown colour (80% coarse - 20% fine). Known estimated thickness reaches about 25-30 cm. The subunit is made

up of about 80% clasts and gravels, 10% blocks and 10% silt-clays. Located in the central pit, current squares N/15-19 of the west stratigraphic profile.

- **Subunit VI-b**: A matrix supported subunit comprising a silt-clayey sediments (70-80% of the total) of dark gray brown colour, with the presence of subangular clasts (major axis between 6-10 cm) and medium limestone gravels (30% coarse - 70% fine). Subunit with presence of macro-fauna. Located in the central pit, current squares N/15-19 of the west stratigraphic profile.

A geochronological examination of the entire stratigraphical sequence of Teixoneres is currently being carried out using luminescence dating. The first processed results provide a final age of 198 ± 11 ka for subunit VI-b (1σ uncertainty range), suggesting an accumulation of the lowermost deposits during MIS 7 or early MIS 6^8^.


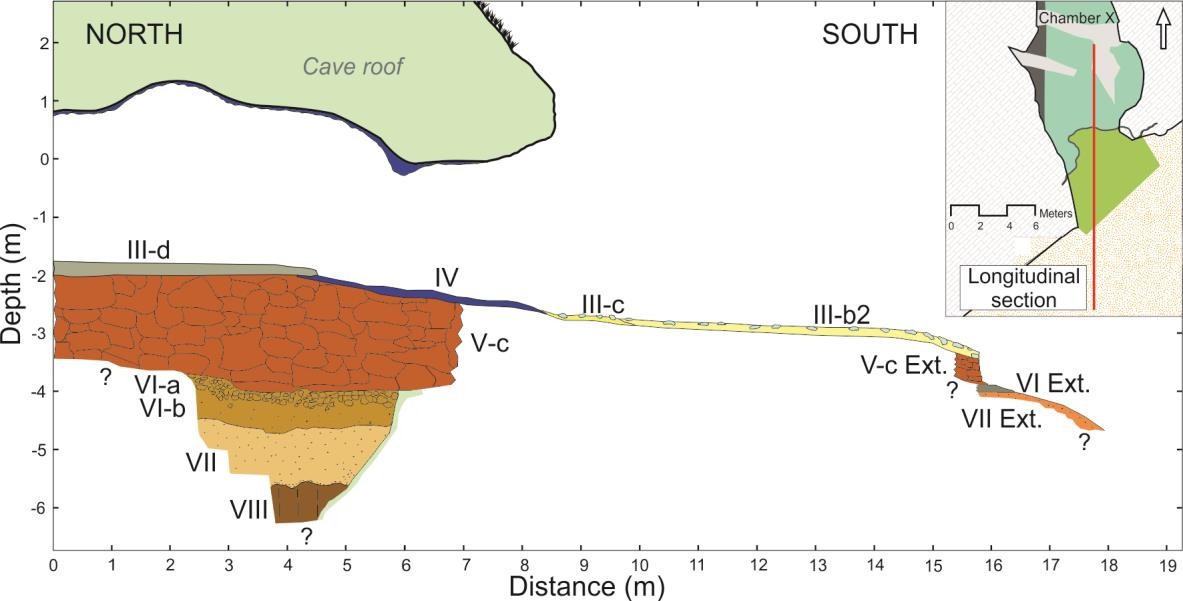


**Figure S2.** Stratigraphic sequence of Chamber X in Teixoneres Cave. Longitudinal section made during 2019 fieldwork.

## Unit V

In this work Unit V is divided into three subunits (Figs. S1 and S2):

- **Subunit V-a**: A localised layer, with variable thickness reaching up to 30 cm. The subunit is made up of angular limestone clasts, whose major axes can reach between 10-15 cm, with a pale brown silty- clayey matrix (10% coarse - 90% fine). No evidence of archaeopaleontological material has been found. Located in the central pit and current squares J-K-L/17 (Fig. S1).
- **Subunit V-b**: A clast supported layer, composed of moderately well-sorted subangular limestone clasts, with major axes reaching 15 cm long and average sizes of approximately 4-8 cm long, and slight dark brown silty-clayey matrix (50% coarse - 50% fine). Its maximum known thickness reaches about 50 cm. Its thickness increases towards the west of the central part of the cavity. This subunit contains archaeological and paleontological remains. Located in the central pit, current squares N/15-19 of the west stratigraphic profile (Fig. S1).
- **Subunit V-c**: The layer is built up by limestone blocks, mostly tabular-subangular blocks that are moderately sorted with major axis sizes varying between 40 and 100 cm long, and a limited matrix made up of loose, pale light brown silts (75% coarse - 25% fine). Its maximum visible thickness reaches ~150 cm and it contains evidence of small and large vertebrates. The origin of this layer must be linked to the fall of blocks from the cave ceiling. Located in the central pit, current squares N/15-19 of the west stratigraphic profile (Figs. S1 and S2).

## Unit IV

Consists of a flowstone with spatially variable thickness. Maximum thickness reaches ~30 cm on the east wall of the cave and decreases towards the central area^1^ (Figs. S1 and S2).

## Unit III

Subunit IIIa was described in Talamo *et al.*^1^ as an homometric monomictic limestone clast supported deposit. In this work we separate it into two subunits (Figs. S1, S2 and S3):

- **Subunit III-a1**: The upper part of this subunit is sharp and irregular^1^. This subunit is made up of tabular subangular limestone blocks up to 30 cm long and up to 10 cm thick, probably originating from

breakdown of the cave ceiling (80% coarse - 20% fine). Located in the central part of Chamber X, current squares I/14-18 of the west stratigraphic profile.

- **Subunit III-a2**: The lower part comprises subangular limestone clasts up to 10-15 cm long, with a slightly cemented, light pale brown grey silt-clay matrix (21% coarse - 79% fine). This subunit contains archaeological and paleontological remains. Located in the central part of Chamber X, current squares I/12- 18 of the west stratigraphic profile.

Subunit IIIb was described in Talamo *et al.*^1^ as an homometric monomictic orthobreccia of limestone cobbles and boulders. In this work we separate it into two subunits (Figs. S1, S2 and S3):

- **Subunit III-b1**: The subunit comprising angular, tabular-equant limestone blocks with thicknesses of 15-20 cm, derived from cave ceiling breakdown. The pores between limestone blocks are filled by a brown silty clay injected upwards from the lower unit (88% coarse - 12% fine). Located in the central part of Chamber X, current squares I/12-17 of the west stratigraphic profile.


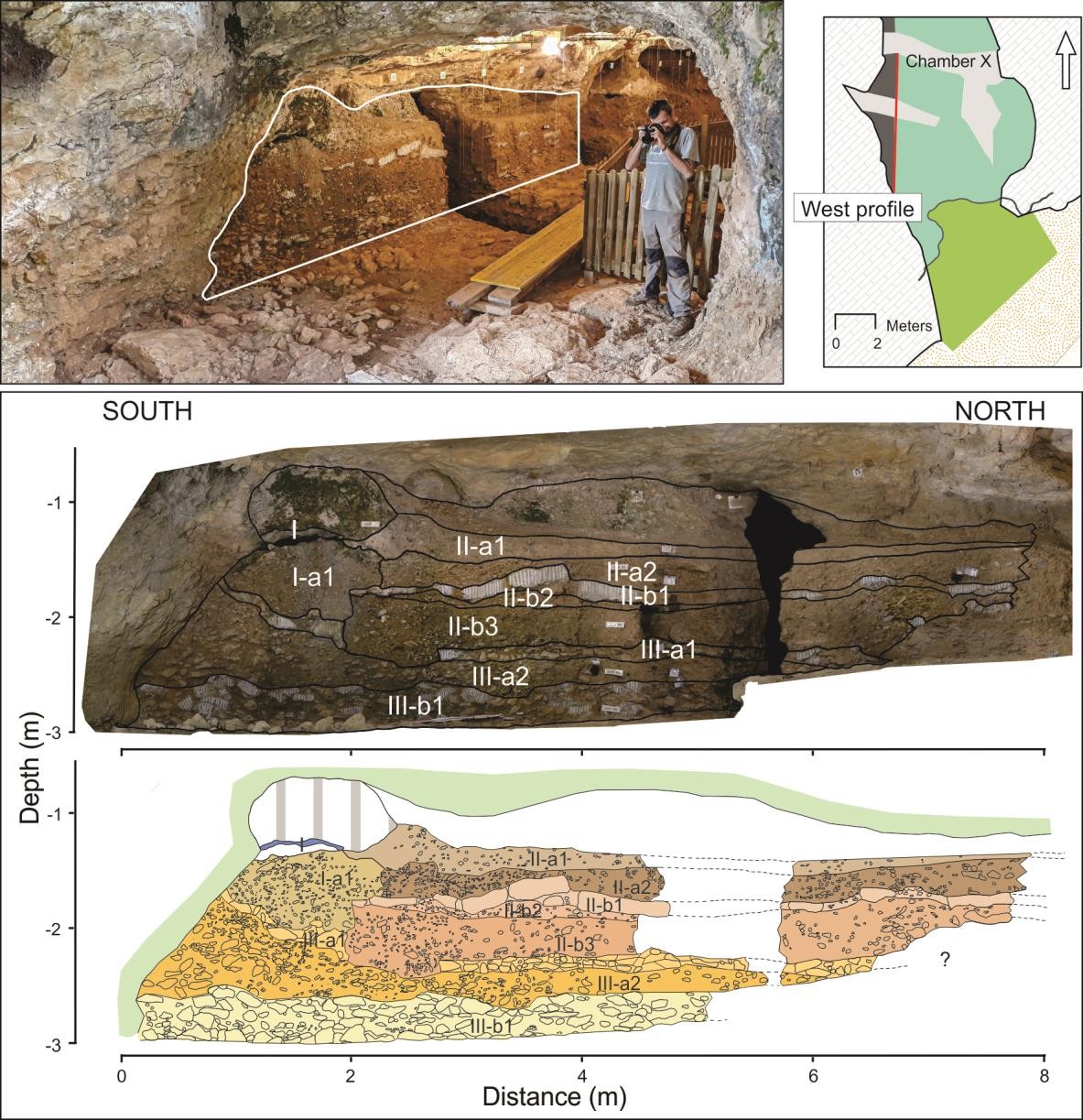


**Figure S3.** Photogrammetry and stratigraphic sequence of West profile of Chamber X in Teixoneres Cave. The photogrammetry was prepared with Agisoft Metashape Professional Version 1.5.5 software.

The images were prepared with the Adobe Photoshop CS5 Version 12.0.4 software.

- **Subunit III-b2**: A poorly sorted subunit, comprises subangular limestone blocks up to 1 m long and clasts that are up to 15 cm long and dark pale brown clay-silt loose matrix (50% coarse - 50% fine). Known estimated thickness reaches about 30-40 cm. Located in the entrance and central part of Chamber X. Extensive excavations have not yet started on this sublevel.

Two other subunits are recognised in Unit III (Fig. S2):

- **Subunit III-c**: A localised, very poorly sorted, matrix supported subunit located along the east wall of the cavity (current squares N-O/11-12), comprises floating equant subangular limestone clasts up to 15 cm long, and light pale yellow silty and loose matrix (50% coarse - 50% fine). It is located in direct contact with Unit IV. Known estimated thickness reaches about 15-20 cm.
- **Subunit III-d**: A poorly sorted subunit, with thickness of up to 30 cm, founded on equant subangular limestone clasts with major longitudinal axes varying between 5-15 cm, and with black cemented silt- clayey matrix (50% coarse - 50% fine). This subunit is located in the inner-central part of Chamber X and on the east stratigraphic profile. Sometimes the subunit is found in direct contact with Unit IV and in other places directly with Unit V. The colour of the matrix is probably related to guano (samples have been taken for further sedimentological analysis).

## Unit II

Subunit II-a was described in Talamo *et al.*^1^ as a homometric monomictic limestone clast orthobreccia. In this work we separate it into two subunits (Figs. S1 and S3):

- **Subunit II-a1**: This subunit is an monomictic orthobreccia made up by limestone subangular clasts ranging from 2 to 4 cm in length long and included in a sparse silty matrix^1^ (8% coarse - 92% fine). This subunit contains archaeological and paleontological remains. Estimated thickness reaches about 15-20 cm. Located in the central part of Chamber X, current squares I/14-19 of the west stratigraphic profile.
- **Subunit II-a2**: A poorly sorted, limestone clast supported subunit. The subangular clasts are 2 to 8 cm long in a sparse light brown loose silty matrix (17% coarse - 83% fine). This subunit contains archaeological and paleontological remains. Estimated thickness is irregular and ranges between 10 and 25 cm. Located in the central part of Chamber X, current squares I/14-19 of the west stratigraphic profile. Subunit II-b was described in Talamo *et al.*^1^ as an homometric monomictic limestone clast supported deposit. In this work we separate it into three subunits (Figs. S1 and S3):
- **Subunit II-b1**: This subunit is made up of tabular subangular limestone blocks up to 50-60 cm long and up to 10-15 cm thick, probably originated from the breakdown of the cave ceiling. The matrix between the blocks is derived from the injection of the lower subunit (85% coarse - 15% fine). Located in the central part of Chamber X, current squares I/14-19 of the west stratigraphic profile.
- **Subunit II-b2**: This is a very local, moderately sorted limestone clast supported subunit, made up of subrounded clasts and cobbles up to 10 cm long and light pale brown silty-clay loose matrix. This subunit is deformed by fallen blocks (14% coarse - 86% fine). Estimated thickness reaches about 10-15 cm. Located in the central part of Chamber X, current squares I/14-17 of the west stratigraphic profile.
- **Subunit II-b3**: A poorly sorted, matrix supported subunit, made up mostly of subangular limestone clasts up to 3-10 cm long, also with a lesser frequency of bigger floating subangular limestone clasts up to 20 cm long, and grey-brown silt-clay matrix and loose matrix (12% coarse - 88% fine). This subunit contains archaeological and paleontological remains. Estimated thickness reaches about 30-40 cm. Located in the central part of Chamber X, current squares I/14-19 of the west stratigraphic profile.

## Unit I

Described by Talamo *et al.*^1^ as a complex of speleothems (flowstone, columns and stalagmites). In this work we have added one new subunit:

- **Subunit I-a1:** A cut-and-fill, channel-like deposit originating from the reactivation of the cave conduit, which shows a coarsening-upward sequence and is clearly eroding subunit II-a1 and II-a2. It is a poorly sorted subunit, made up mostly by subangular limestone clasts up to 3-10 cm long, and a brown, silt-clay, loose matrix (18% coarse - 82% fine). Estimated thickness reaches about 40-50 cm. Located in the central part of Chamber X, current square I/13 of the west stratigraphic profile (Fig. S3).

During the 2019 fieldwork in the external sector of the cave we identified three new layers (squares M- P/2-5; Fig. S2):

- **Subunit V-c Ext.**: Subunit formed from mostly tabular subangular limestone blocks, moderately sorted with major axis sizes varying between 10 cm and 25 cm long, and with a sparse matrix comprising loose silts of pale light brown colour. Known estimated thickness reaches about 50 cm.
- **Unit VI Ext.**: A matrix supported unit forming a silt-clayey level (70-80% of the total) of dark gray brown colour, with the presence of subangular clasts (major axis size is between 6-10 cm) and medium limestone gravels. Known estimated thickness reaches about 10-15 cm.
- **Unit VII Ext.**: The unit comprises sands and subangular limestone gravels with a red-pale silty well- cemented matrix. Known estimated thickness reaches about 40-50 cm.

Due to the stratigraphic position and characteristics of the aforementioned units, we have decided to undertake a first-order correlation with the internal units. However, our plan is to improve and define this correlation with greater precision over future excavation seasons.

# Stratigraphic analysis

The stratigraphic studies performed at Teixoneres have allowed us to better delimit and describe the units and subunits that comprise the deposit. The results have permitted us to conduct a deeper investigation into the lithostratigraphic sequence of Chamber X, as well as an improved understanding of the site formation processes.

The sedimentary infilling of Teixoneres Cave is principally composed of two big members: 1- a lower member of allochthonous facies, which are remnant deposits of alluvial sediments related to the Torrent del Mal river activity; and 2- an upper member composed of autochthonous facies deposits originating from the breakdown of the cave ceiling and walls, the gradual retreat of the cave roof at the entrance, and from fine sediment mostly originating from in situ weathering, debris flows and infiltration.

The sedimentary characteristics of the lower member (Units VII and VIII) in the central inner part of the cave (laminated clays) suggests deposition from suspension during poorly drained periods, consistent with very low sediment supply and a marshy environment. In contrast, the alteration of these layers with clayey laminated sands indicates paleoenvironmental changes from fine distal to low energy proximal floodplain deposits, which is common in overbank areas^9^. Furthermore, the presence of iron-manganese oxide accumulations in this member suggests a water-saturated, reducing paleoenvironment during deposition^10^. In sum, the sediments of the lower member indicate low energy flow conditions in floodplain or abandoned channel areas, which alternated with periods of shallow and still waters conditions in a reducing environment.

The upper member (Units I to VI) is composed of limestone blocks and clasts, fine sediments and speleothems. Limestone blocks and clasts are located at the entrance and in the central inner part of the cave, and comprises between 80% and 100% of the sediment by volume in subunits II-b1, III-a1, III-b1, V- a, and V-c. In general, the gaps between the limestone blocks and clasts in these subunits are filled by a silty matrix. Moreover, subunits I-a1, II-a1, II-a2, II-b2, II-b3, III-a2, III-b2, III-c, III-d, VI-a, and VI-b are composed of floating limestone blocks, small fragmented clasts and gravels in poorly sorted matrices. Finally, Units I and IV are secondary carbonate speleothems formed by the precipitation of calcite from waters that had percolated through the adjacent carbonate bedrock^11^.

The sedimentary infill of Teixoneres Cave, which is composed mainly of angular-subangular bedrock blocks and clasts, could have originated from a wide range of geological processes that have affected this Mediterranean upland region, e.g. cryoclastism, dissolution, seismic activity and hydration shattering^12–15^. Though some authors do not recommend the widespread use of cave infill deposits as climatic indicators for paleoenvironmental reconstructions^15–17^, there have been studies relating these types of deposits to climate-related cryoclastism processes (breakdown of cave walls and ceilings)^12,17^. In our case, following the defined criterion by Mallol *et al.*^18^, the units composed of well-sorted, angular blocks and clasts could be derived from cryoclastic breakdown, leading us to contemplate that formation of subunits II-b1, III-a1, III-b1, V-a, and V-c could be associated with periods of extreme cold climate. The chronology of subunits II-b1, III-a1, and III-b1 possible correspond to the final part of the MIS 4 through to the end of MIS 3, a cold period characterized by intense and short climatic fluctuations^19^, which would be in agreement with the cold adapted taxa recovered from subunits II-a, III-a and III-b^20^. This interpretation is also consistent with other similar cave infill deposits studied in the Mediterranean and Iberian Peninsula^12,17,21–23^. Furthermore, subunits II-b1, III-a1, and III-b1 exhibit evidence of periodic accumulation, alternating between coarse and fine sediment deposition, which could indicate a potential climatic signature. It is worth keeping in mind, however, that a tectonic origin for these deposits cannot be excluded because, despite being a region of low historical seismicity, there is evidence of moderate magnitude earthquakes affecting the Catalan coastal ranges during the Middle-Late Pleistocene^24,25^.

The fine sediments accumulated at Teixoneres Cave are poorly sorted and massive (e.g. not laminated). The origin of such fine sediments in cave contexts could be related to a variety of processes and sources, including fluvial, aeolian, debris flow, infiltration and in situ weathering. Each of these processes has its own sedimentary characteristics, and can be summarised as follows: 1- allochthonous fluvial sediments usually show laminations, roundness and lack of sorting; 2- allochthonous aeolian sediments are usually finely laminated, and not well sorted; 3- debris flow sediments are typically composed of floating coarse deposits in a poorly sorted silty matrix; 4- infiltration sediments, which are transported through bedrock joints and crevices, and; 5- in situ autochthonous weathering of corroded clasts and gravels^23^. Following these criteria, our initial and preliminary interpretations of the fine grain sediments at Teixoneres indicate that they could have originated from a combination of debris flow and in situ weathering processes, which may have been caused by reactivation of the cave conduit, as observed in subunit I-a1. However, it should be noted that for further analysis is required to reach a more definitive understanding of the fine sediment formation processes, preferably with the application of sedimentary and micromorphological techniques.

# SUPPLEMENTARY INFORMATION – section 2

**Spatial analysis results**


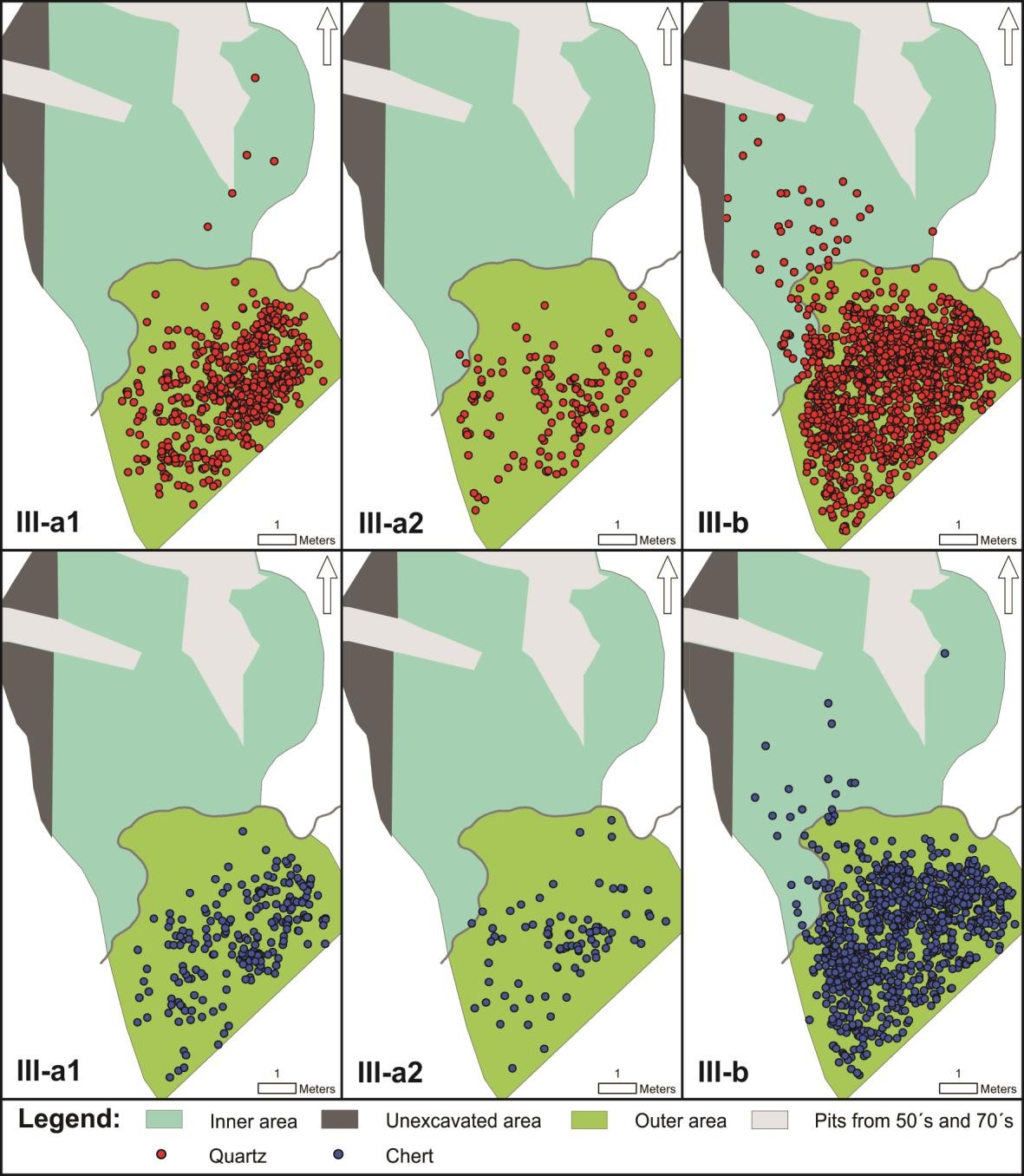


**Figure S4**. Spatial distribution of quartz and chert lithic artefacts in subunits III-a1, III-a2 and III-b of Teixoneres Cave.


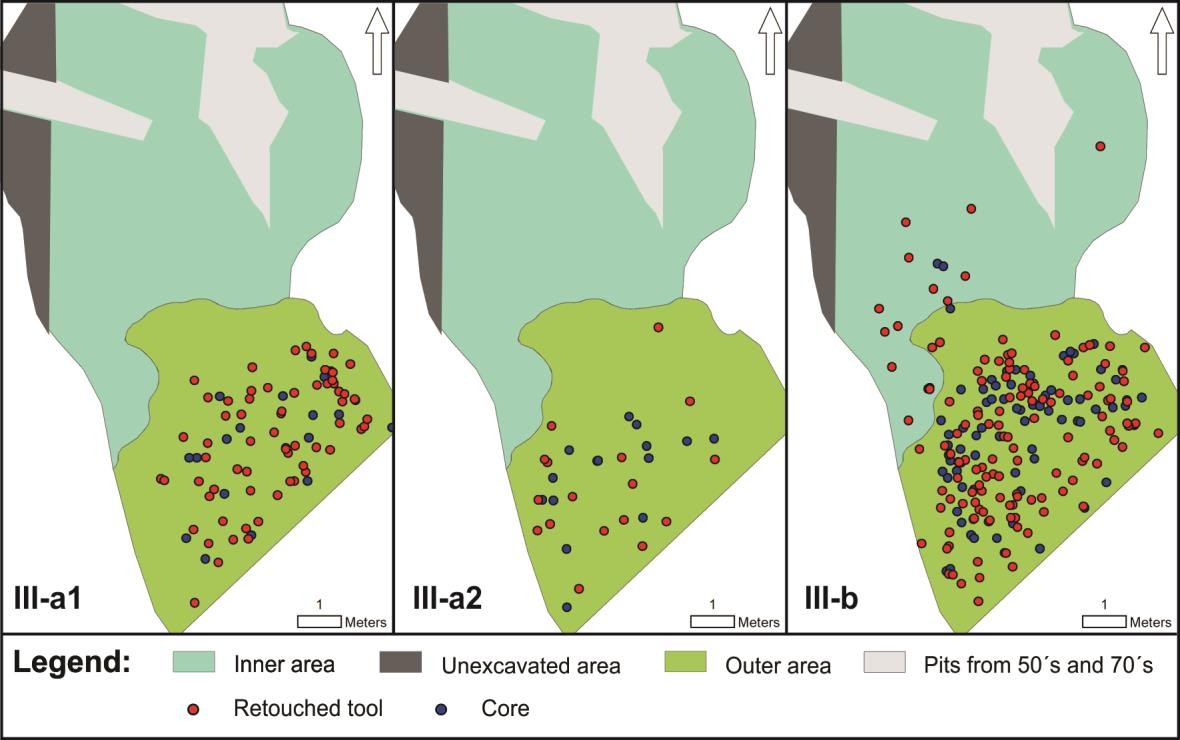


**Figure S5.** Spatial distribution of tools and cores in subunits III-a1, III-a2 and III-b of Teixoneres Cave.

| **Tools** | **Subunit** | | |
| --- | --- | --- | --- |
|  | **III-a1** | **III-a2** | **III-b** |
| Scraper | 17 | 7 | 49 |
| Double scraper | 1 | - | 1 |
| Point | 9 | 2 | 13 |
| Mousterian point | 4 | 2 | 17 |
| Convergent tool | 3 | - | 3 |
| Notched tool | 8 | 3 | 10 |
| Denticulate | 14 | 2 | 28 |
| Fragment | 9 | 1 | 12 |
| **Total** | 65 | 17 | 133 |

**Table S1.** Retouched tools in subunits III-a1, III-a2 and III-b of Teixoneres Cave.

| **Cores** | **Subunit** | | |
| --- | --- | --- | --- |
|  | **III-a1** | **III-a2** | **III-b** |
| Levallois pref. | 1 | - | - |
| Levallois rec. uni. | - | - | 1 |
| Levallois rec. centr. | 2 | - | - |
| Hierarchized centr. | - | - | 4 |
| Discoid | - | - | 2 |
| Unidirectional | 3 | 3 | 11 |
| Bidirectional | - | - | 3 |
| Centripetal | 1 | 1 | 9 |
| Bipolar | 2 | - | 1 |
| Poliedric | - | - | 1 |
| Core-on-flake | 7 | 6 | 14 |
| Core fragment | 3 | 6 | 29 |
| **Total** | 19 | 16 | 75 |

**Table S2.** Cores in subunits III-a1, III-a2 and III-b of Teixoneres Cave.


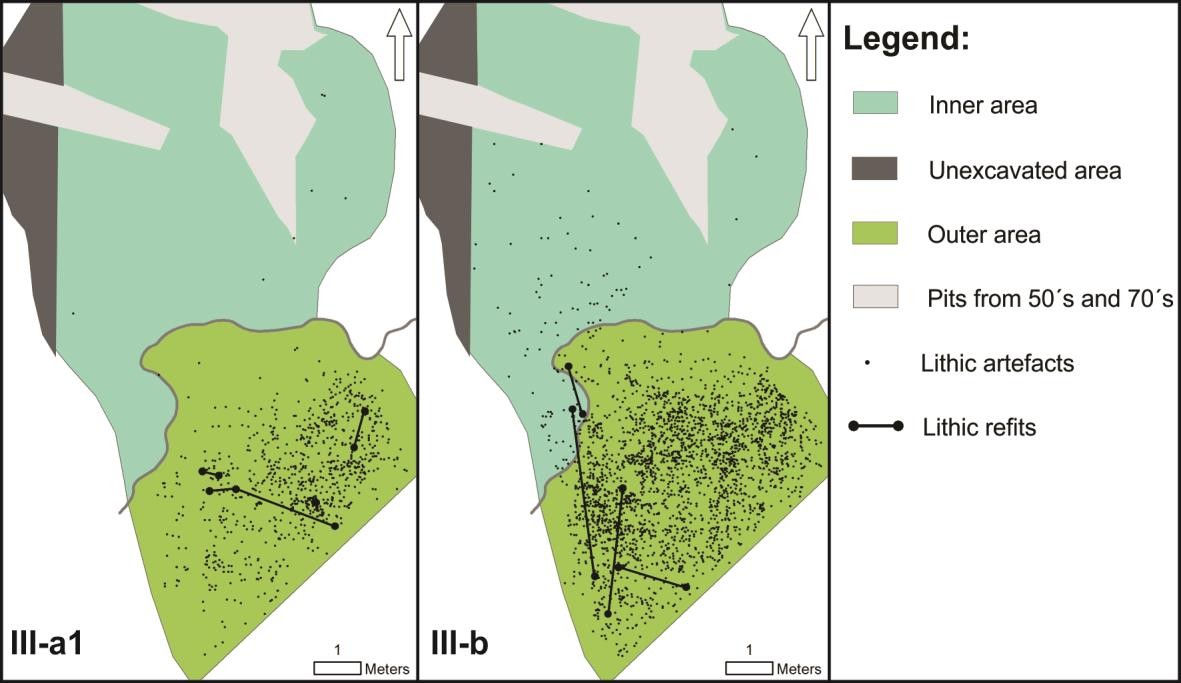


**Figure S6**. Spatial distribution of lithic refits.

| **Subunit** | **Category** | **Raw material** | **Distance (cm)** |
| --- | --- | --- | --- |
| III-a1 III-a1  III-a1 | Core fragment Core fragment  Core fragment | Chert Chert  Chert | 225.30  56.14 |
| III-a1  III-a1 | Flake Core | Quarzite Quarzite | 36.13 |
| III-a1  III-a1 | Tool Tool | Chert Chert | 80.36 |
| III-a1  III-a1 | Flake Flake | Chert Chert | 143.45 |
| III-b  III-b | Flake Flake | Chert Chert | 323.14 |
| III-b  III-b | Flake Flake | Chert Chert | 150.28 |
| III-b  III-b | Flake Flake | Chert Chert | 105.36 |
| III-b  III-b | Flake Core | Quartz Quartz | 269.78 |

**Table S3.** Characteristics of lithic refits.


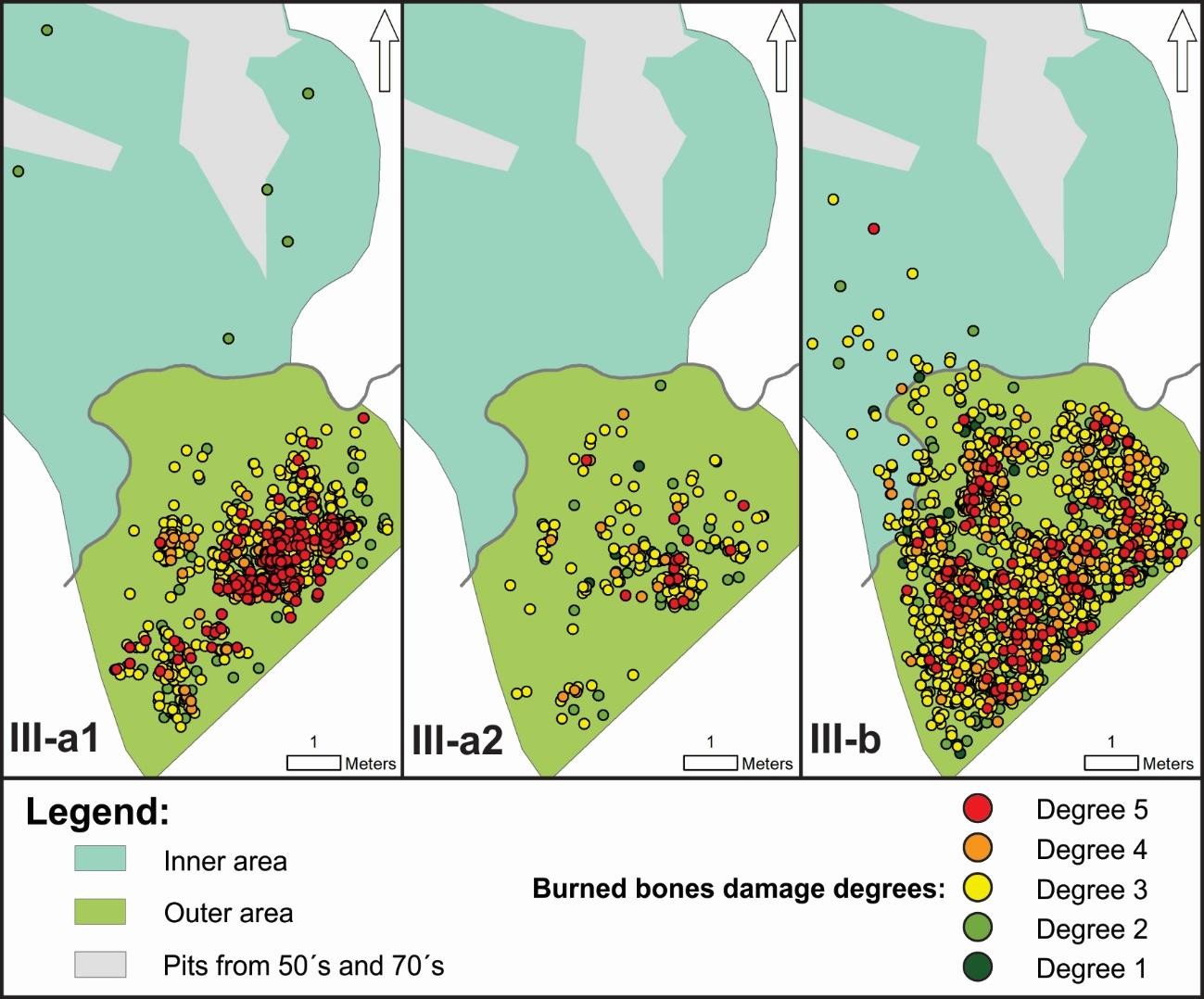


**Figure S7**. Spatial distribution of burned bone specimens from subunits III-a1, III-a2 and III-b of Teixoneres Cave following the classification in degrees suggested by Stiner *et al*. ^26^.


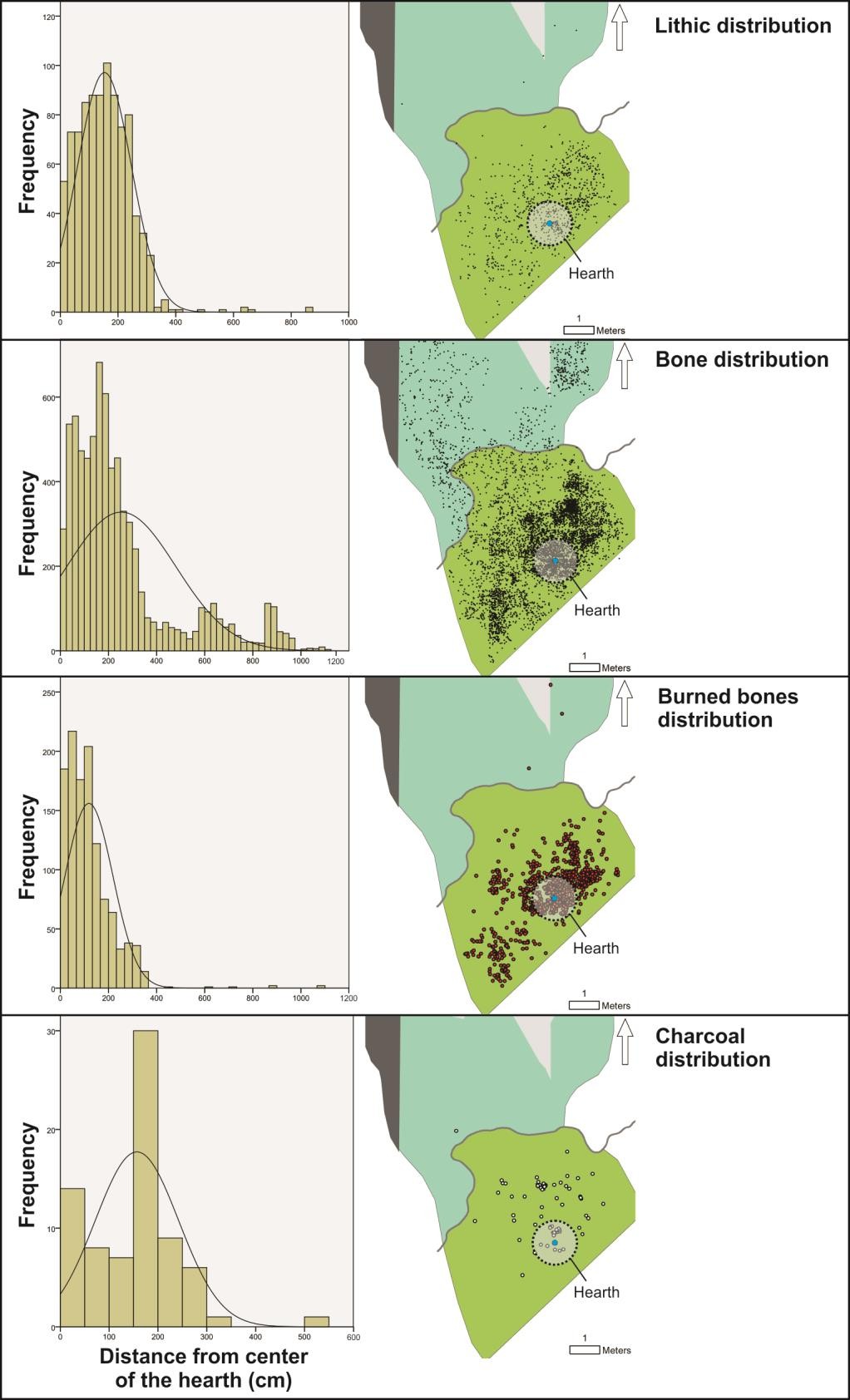


**Figure S8**. Spatial distribution of lithics, bones, burned bones and charcoal fragments and distance- decay curves taking into account a central point from the main hearth identified in subunit III-a1 of Teixoneres Cave.


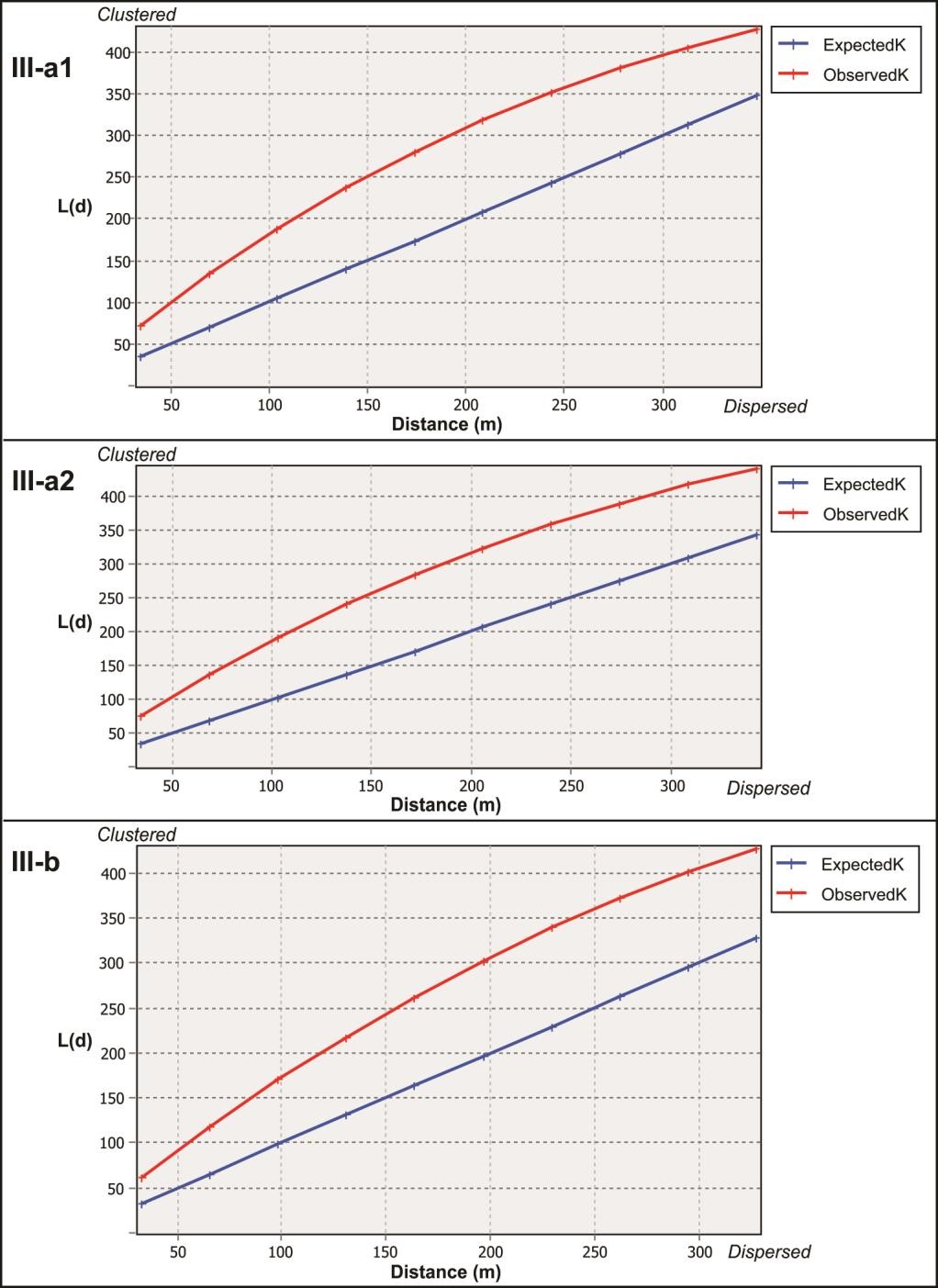


**Figure S9**. Ripley's K-function for all archaeological materials recovered in subunits III-a1, III-a2, and III-b.

# References

1. Tálamo, S. *et al.* The Radiocarbon Approach to Neanderthals in a Carnivore Den Site: a Well- Defined Chronology for Teixoneres Cave (Moià, Barcelona, Spain). *Radiocarbon* **58**, 247–265 (2016).
2. Canals, A., Vallverdú, J. & Carbonell, E. New archaeo-stratigraphic data for the TD6 level in relation to Homo antecessor (Lower Pleistocene) at the site of Atapuerca, North-central Spain. *Geoarchaeology* **18** (5), 481–504 (2003).
3. Sánchez-Romero, L. *et al*. Breaking the palimpsest: an approach to the cultural sequence of Neanderthal occupation at the Navalmaíllo rockshelter, Pinilla del Valle (Spain). *Trab. Prehist.* **74**(2), 225–237 (2017).
4. Vaquero, M. & Pastó, I. The definition of spatial units in Middle Palaeolithic sites: the hearth- related assemblages. *J. Archaeol. Sci.* **28**(11), 1209–1220 (2001).
5. Stein, J. K., Deo, J. N., and Phillips, L. S. Big sites-short time: accumulation rates in archaeological sites. *J. Archaeol. Sci*., **30**(3), 297–316 (2003).
6. Roy Sunyer, M., Roda Gilabert, X., Benito-Calvo, A., Martínez-Moreno, J. & Mora Torcal, R. Verificando la integridad del registro arqueológico: análisis de fábricas en las unidades arqueológicas del paleolítico medio/superior de la Cova Gran (Santa Linya, Lleida). *Treballs d'arqueologia* **20**, 55–77 (2014).
7. Serrat, D. & Albert, J. F. Estudio sedimentológico de los materiales de relleno de la Cova de les Teixoneres. *Speleon* **20**, 63–70 (1973).
8. Ochando, J. *et al.* Neanderthals in a highly diverse, Mediterranean-Eurosiberian forest ecotone: the Pleistocene pollen records of Teixoneres Cave, northeastern Spain. *Quat. Sci. Rev*.<http://dx.doi.org/10.1016/j.quascirev.2020.106429>(2020).
9. Mial, D. *The geology of fluvial deposits* (Springer, Berlin-Heidelberg, 2006).
10. Angelucci, D. *et al.* Formation processes at a high resolution Middle Paleolithic site: Cueva Antón (Murcia, Spain). *Quat. Int.* **315**, 24–41 (2013).
11. Bradley, R. S. *Paleoclimatology. Reconstructing climates of the Quaternary* (Third Edition) (Academic Press, Oxford, 2015).
12. Laville, H. Deposits in calcareous rock shelters: Analytical methods and climatic interpretation. In *Geoarchaeology* (eds. Davidson, D. A. & Shackley, M. I.), 137–155 (Duckworth, London, 1976).
13. Collcutt, S. N. The analysis of Quaternary cave sediments. *World Archaeol.* **10**, 290–301 (1979).
14. Farrand, W. R. Rockshelter and cave sediments. In *Archaeological sediments in context* (eds. Stein, J. K. & Farrand, W. R.), 21–39 (Center for the Study of Early Man, Orono, 1985).
15. Woodward, J. C. & Goldberg, P. The sedimentary records in Mediterranean rockshelters and caves: Archives of environmental change. *Geoarchaeology* **16**, 465–466 (2001).
16. Bailey, G. N. & Woodward, J. C. The Klithi deposits: sedimentology, stratigraphy and chronology. In *Klithi: Palaeolithic settlement and Quaternary landscapes in northwest Greece. Vol. 1: Klithi excavations and intra-site analysis* (ed. Bailey, G. N.), 61–94 (McDonald Institute for Archaeological Research, Cambridge, 1997).
17. Courty, M. A. & Vallverdú, J. The microstratigraphic record of abrupt climate changes in cave sediments of the Western Mediterranean. *Geoarchaeology* **16**, 467–500 (2001).
18. Mallol, C., Hernandez, C. M., & Machado, J. The significance of stratigraphic discontinuities in Iberian Middle to Upper Paleolithic transitional sites. *Quat. Int.* **275**, 4–13 (2012).
19. Barron, E. & Pollard, D. High-Resolution Climate Simulations of Oxygen Isotope Stage 3 in Europe 1. *Quat. Res*. **58**(3), 296–309 (2002).
20. Álvarez-Lao, D. J., Rivals, F., Sánchez-Hernández, C., Blasco, R. & Rosell, J. Ungulates from Teixoneres Cave (Moià, Barcelona, Spain): Presence of cold-adapted elements in NE Iberia during the MIS 3. *Palaeogeogr. Palaeoclimatol. Palaeoecol*. **466**, 287–302 (2017).
21. Woodward, J. C. & Bailey, G. N. Sediment sources and terminal Pleistocene geomorphological process recorded in rockshelter sequences in northwest Greece. In *Tracers in Geomorphology* (ed. Foster, I. D. L.) 521–551 (John Wiley, Chichester, 2000).
22. Goldberg, P., Laville, H. & Meignen, L. Stratigraphy and geoarchaeological history of Kebara Cave, Mount Carmel. In *Kebara Cave* (eds. Bar Yosef, O. & Meignen, L.), Part 1, 49–89 (Peabody Museum of Archaeology and Ethnology, Harvard University, Cambridge, 2007).
23. Benedetti, M. M., Haws, J. A., Bicho, N. F., Frield, L., & Elwood, B. B. Late Pleistocene site formation and paleoclimate at Lapa do Picareiro, Portugal. *Geoarchaeology* **34**, 698–726 (2019).
24. Masana, E. Evidence for past earthquakes in an area of low historical seismicity: the Catalan coastal ranges, NE Spain. *Annali di Geofisica* **39**, 689–704 (1996).
25. Masana, E., Villamarín, J.A., Sánchez Cabañero, J., Plaza, J. & Santanach, P. Seismogenic faulting in an area of low seismic activity: Paleoseismicity of the El Camp fault (Northeast Spain). *Netherlands J. Geosci./Geol. Mijnbouw* **80**(3–4): 229–241 (2001).
26. Stiner, M. C., Kuhn, S. L., Weiner, S. & Bar-Yosef, O. Differential burning, recrystallization, and fragmentation of archaeological bone. *J. Archaeol. Sci*. **22**(2), 223–237 (1995).
